# Exact concentration regions for stimulus-switched competitive binding under finite depletion

**DOI:** 10.1039/d6ra06172a

**Published:** 2026-10-07

**Authors:** Hyun Seo

**Affiliations:** a Independent Researcher Seoul Republic of Korea leviseo2019@gmail.com

## Abstract

Transferring binary binding constants into competitive mixtures is a common but rarely validated step in stimulus-programmed molecular recognition. A pH-programmed Co_8_L_12_ coordination cage reported by Cullen *et al.*, *Chem. Sci.*, 2015, **6**, 4025–4028 supplies a parameter-free test because its reported C/B composition at pH 7.5 requires *K*^eff^_B_(7.5) < 6.39 M^−1^, yet the entire rectangle formed from the quoted marginal affinity ranges lies above this bound, with the most favourable point still giving 8.78 M^−1^. This study develops a method that accounts for finite depletion, which separates concentration-window limitations from parameter-transfer failure. For two guests with one total fixed, monotonicity converts independent fidelity and occupancy targets into one-sided thresholds whose intersection is the complete admissible interval. A pairwise endpoint bound extends the consistency test to mixtures with additional same-site competitors, and a free-host-parametrized boundary construction maps the admissible region when two totals vary independently. For the Co_8_L_12_ cage reported by Cullen *et al.*, *Chem. Sci.*, 2015, **6**, 4025–4028, a reference model neglecting depletion predicts a controlled-free-concentration reference of 95.95%, while the exact finite-depletion maximin is restricted to 94.36%. Conversely, a pH-responsive naphthotube operating with a wider selectivity margin retains a broad 95% target window and requires only a minor 0.22 percentage-point adjustment for depletion effects. The method therefore identifies when finite depletion changes a design decision and when transferred binary constants are inconsistent with competitive-mixture observations.

## Abstract 2

Exact finite-depletion intervals distinguish feasible concentration windows from empty ones: a 95% handoff is inaccessible in the Cullen binary projection but retains a broad window for the naphthotube.

## Graphical abstract

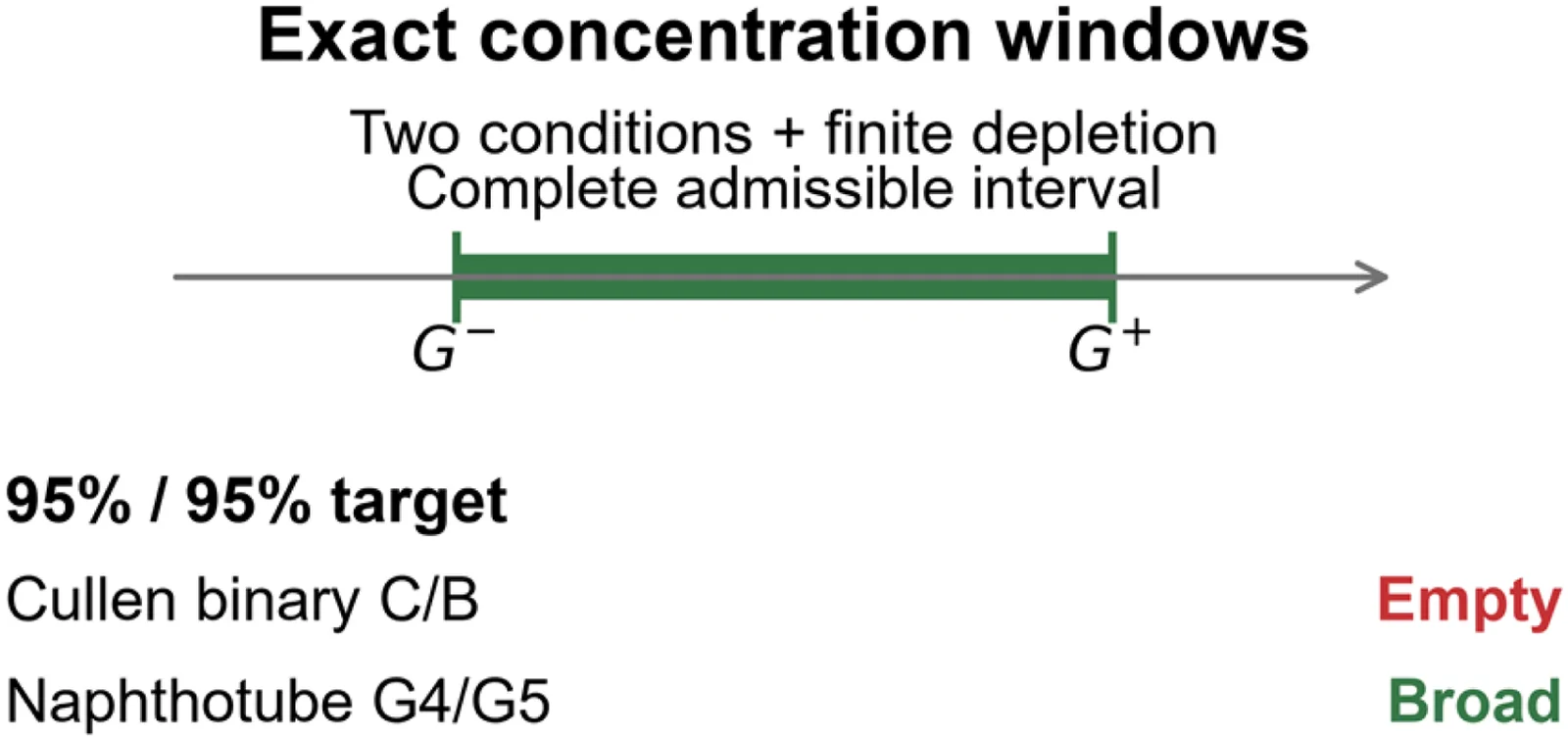

## 1 Introduction

A performance claim for a competitive selector can be tested against its own reported constants, with no fitted parameters, and may still fail. The pH-programmed Co_8_L_12_ cage of Cullen and co-workers^1,2^ is such a case: the reported mixture yields more than 97% cage-C and less than 2% cage-B, yet applying the reported binding constants in an ideal same-site model cannot reproduce that C/B ratio. The observation constrains *K*^eff^_B_(7.5) < 6.38885 M^−1^, whereas the same-site model gives *K*^eff^_B_(7.5) = 8.78325 M^−1^ even at the most favourable corner of the quoted uncertainties. This mismatch reflects the non-transferability of a minimal parameterization, not a failure of the experimental selector. The same formalism raises a separate design question: what total B concentration could satisfy a two-condition C → B handoff under a specified model? Equivalently, how much competing guest is needed to achieve the specified change in host preference between the two conditions?

The field of molecular recognition developed from macrocyclic host-guest chemistry and concepts such as preorganization, complementary structures, and programmed supramolecular self-assembly,^3–7^ and metal-mediated self-assembly extended these concepts to coordination cages with enclosed guest-binding cavities.^8,9^ Guest binding to and release from these cages can be controlled by external stimuli, such as solvent, light, temperature, and photochemical transitions,^10–13^ while quantitative studies have shown that both binding thermodynamics and exchange kinetics are essential factors in these processes.^14^

Association constants and stoichiometry are generally determined by methods such as spectroscopic titrations, calorimetry and model fitting. The reliability of these estimates depends on the concentration ranges used, the chosen binding model, whether the parameters can be uniquely identified and how uncertainty is treated.^15–21^ Chemical speciation methods generally solve coupled sets of mass-action equilibrium equations from spectroscopic or potentiometric titration measurements.^22–25^

Designing molecular recognition that responds to external stimuli presents both design and formulation challenges for typical equilibrium modelling. For a two-condition molecular switch, target purity (composition) and host occupancy must be specified independently, because high conditional selectivity can coexist with poor host occupancy. The mixture must contain enough competitor to dominate at one condition without compromising selectivity at the other. The practical problem therefore concerns both the behaviour of a selected mixture and the range of component concentrations that satisfies all target requirements.

Finite depletion is a well-known feature in tight-binding systems. Studies on high-affinity receptors and tight-binding enzymes have shown that when the receptor and ligand are present at similar concentrations, free and total concentrations cannot be used interchangeably.^26–30^ One-condition competitive binding theory includes the Cheng–Prusoff relationship, logistic response models, kinetic competition models, mathematical corrections, and assay-validation models.^31–37^ Additionally, numerical algorithms are available to solve for much larger ligand pools and support affinity optimization.^38^

Related theories range from the Langmuir single-site isotherm to mixture models and thermodynamic adsorption frameworks.^39–41^ Explicit analytical solutions for two-component finite-batch Langmuir equilibrium were also recently developed.^42^ Separation theory provides a broader context for how selectivity limits single-stage separation.^43,44^ These studies mostly focus on predicting, fitting, or interpreting conditions for a given set of inputs, forming the base of the present formulation problem (Table 1).

**Table 1:** Closest analytical antecedents and the bounded inverse-design distinction

Work	Established result	Distinction of present contribution
Tight-binding/depletion literature^26–28^	Finite-total mass balances and receptor/ligand depletion	No bounded two-condition inverse specification
Wang (1995)^29^	Exact forward two-ligand total-concentration equilibrium	No bounded two-condition specification
Competitive adsorption^39,40,42^	Mixture isotherms and analytical finite-batch two-component solution	No fidelity/occupancy formulation window
Blay *et al.* (2020)^38^	Robust numerical many-ligand equilibrium and optimization	Numerical forward/optimization workflow
Present work	Closed-form bounded inverse specification	Complete fixed-one-total interval plus consistency bound

This work addresses a more specific design problem. The objective is to find total guest concentration intervals that satisfy independent fidelity and host occupancy targets at two conditions. The desired output is either the complete set of admissible concentrations, or a proof that no such set exists. A finite numerical grid can identify feasible samples, but completeness requires additional mathematical information; numerical threshold searches combined with monotonicity can also recover the full interval to a specified tolerance. Furthermore, simplified models based on unbound concentrations may fail to provide reliable solutions in finite closed systems because depletion changes the unbound guest ratio differently at the two conditions.

This work provides a closed-form solution to the bounded inverse-design problem for the finite-depletion one-site model. Specifically, it introduces: (i) an odds-product representation with initial endpoint screening bounds; (ii) a sufficient range of total concentration ratios for asymmetric fidelity targets; (iii) explicit thresholds for selectivity and host occupancy whose intersection defines the complete formulation window when one total concentration is fixed; and (iv) a pairwise consistency limit applicable to mixtures containing any number of additional competing species. A two-variable extension, where the solution curve is replaced by exact boundary curves parametrized by the free-host concentration, is also derived, enabling the mapping of concentration regions when two guest totals are varied independently, without requiring assumptions about convexity or a single-interval topology. This method is applied to the pH-regulated coordination cage presented in the study by Cullen *et al.*,^1,2^ including the full three-guest concentration plane and a study on pH-responsive naphthotube systems by Wang *et al.*,^45^ which reports a two-state pH switch with association constants for both guests at both pH values.

## 2 Model, metrics and the known forward problem

Consider one ideal, mutually exclusive 1 : 1 host site and two guests *i* and *j*. At condition *x* ∈ {*a*, *b*}, the association constants are *K*_*i*_(*x*), *K*_*j*_(*x*) > 0, the guest totals are *G*_*i*,T_, *G*_*j*,T_ > 0, the host total is *H*_T_ > 0, and *h*_*x*_ is the free-host concentration. The bound concentrations are1
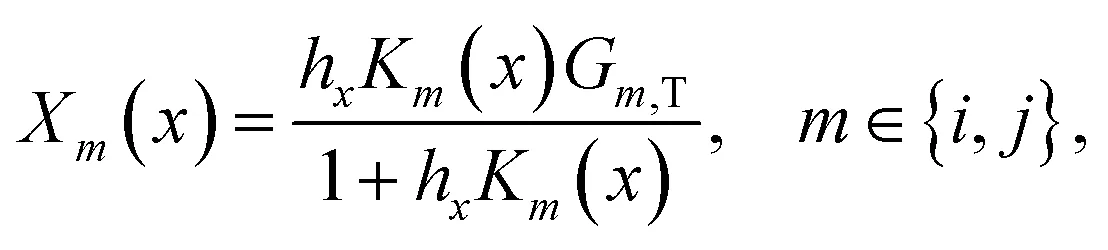
with

2 *H*_T_ = *h*_*x*_ + *X*_*i*_(*x*) + *X*_*j*_(*x*).

The same finite-total mass-balance structure is standard in tight-binding and receptor-depletion analyses and can also be handled by general chemical-speciation methods.^22,24,26–29^ The physical root is unique because the host-balance function is strictly increasing. This is the established forward problem; exact two-component and general numerical treatments are available.^29,38,42^

Three metrics are kept distinct:3
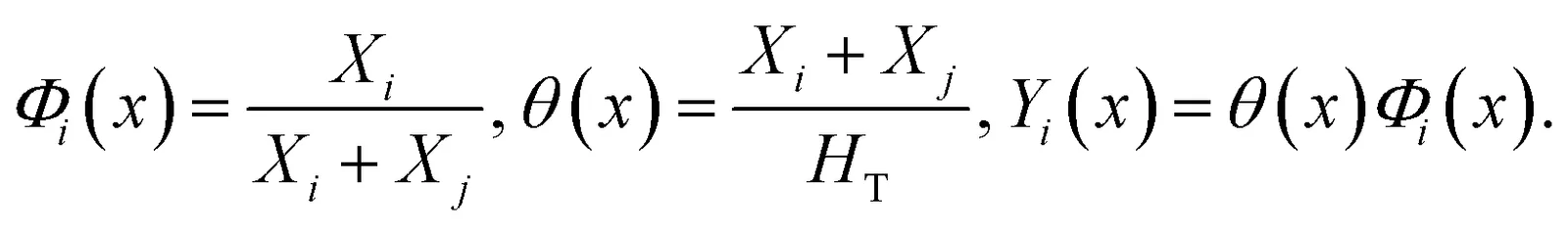


They are conditional bound-state fidelity, total host occupancy and desired-complex yield, respectively. All exact, necessary-and-sufficient and impossibility statements below are conditional on this ideal concentration-based model and fixed condition-specific constants.

The applicable scope is an equilibrated host population represented by one mutually exclusive binding site with fixed affinities at each condition. Multiple non-equivalent sites, simultaneous binding of several guests, cooperativity or coupled conformational equilibria require additional populations and mass balances. A conformationally responsive host can use effective constants here only if the stated one-site model describes its equilibrium behaviour over the concentration range considered; otherwise the additional states and the relevant monotonicity must be treated explicitly. The present equations do not predict switching rates, kinetic trapping or hysteresis. An empty interval excludes a specification within the declared model and parameters, rather than all possible implementations of the chemical switch.

Fidelity describes which guest occupies the host among occupied hosts; occupancy describes how much of the total host is occupied. Thus 95% fidelity at 20% occupancy gives a desired-complex yield of only 0.95 × 0.20 = 19% of the total host. The two targets must be specified independently. A symbol table with units and practical meanings is provided in SI Section 1.

For comparison with a non-depleting reference, let *r* = [*i*]_free_/[*j*]_free_ under ideality and define4
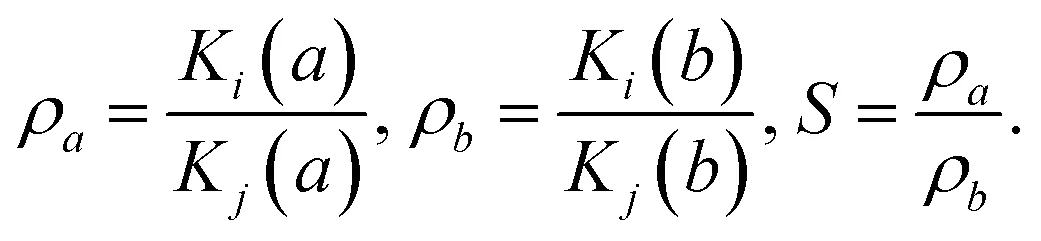


Controlled free concentrations give *O*_*a*_ = *rρ*_*a*_ and *O*_*b*_=(*rρ*_*b*_)^−1^, where *O* = *Φ*/(1 − *Φ*). Equalization gives *r*_0_=(*ρ*_*a*_*ρ*_*b*_)^−1/2^ and the elementary reference maximin 
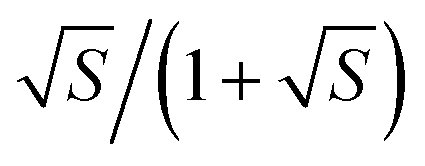
. Separation theory provides the broader conceptual setting of selectivity-limited single-stage performance,^43,44^ but no specific attribution of this host–guest derivation is required. In a finite closed sample, the total ratio *R* = *G*_*i*,T_/*G*_*j*,T_ generally differs from *r*.

## 3 Two-condition screening bounds

Define5
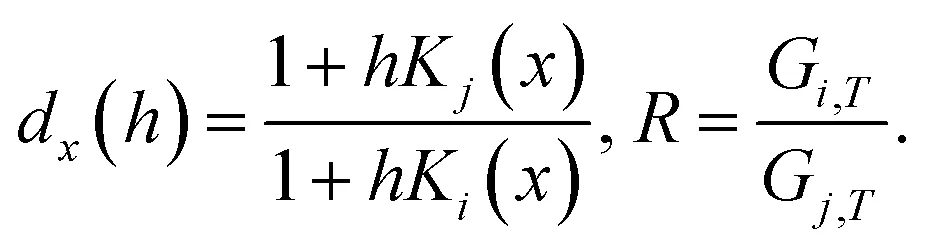


The target odds are6
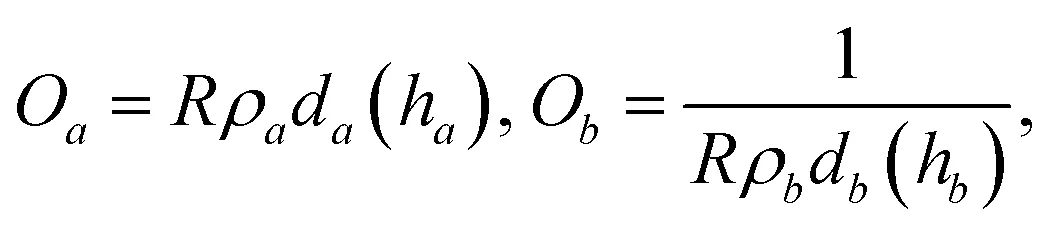
so7
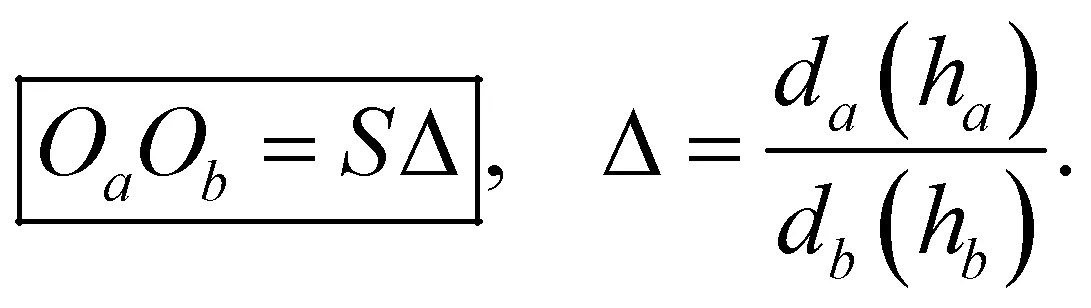


Each *d*_*x*_ is monotone on 0 ≤ *h* ≤ *H*_T_. With

8 *d*_*x*_^−^ = min{1,*d*_*x*_(*H*_T_)}, *d*_*x*_^+^ = max{1,*d*_*x*_(*H*_T_)},

the bounds are9
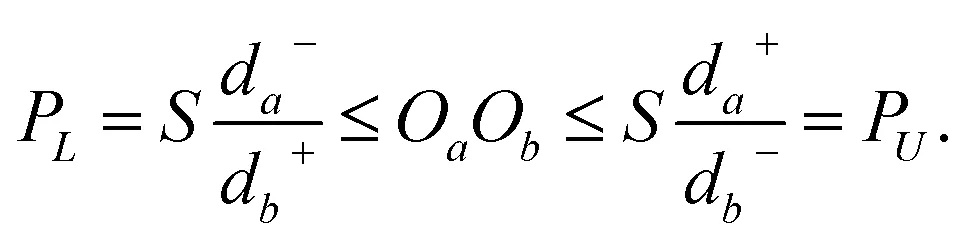


Let the independent target fidelities be 0 < *η*_*a*_, *η*_*b*_ < 1, with odds10
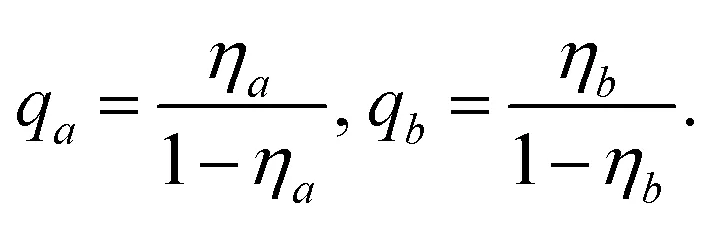


Because simultaneous satisfaction requires *O*_*a*_*O*_*b*_ ≥ *q*_*a*_*q*_*b*_, the condition11
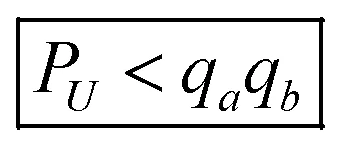
proves global impossibility within the ideal model.

The odds *q* = *η*/(1 − *η*) express the required target/non-target ratio: 95% fidelity corresponds to 19 : 1. The upper product bound asks whether even the most favourable depletion correction can provide the combined odds at both conditions. Passing this necessary screen does not establish feasibility. The endpoints *h* = 0 and *h* = *H*_T_ are bounding limits, not two assumed experimental equilibria.

A complementary sufficient result gives a full interval of admissible ratios:12
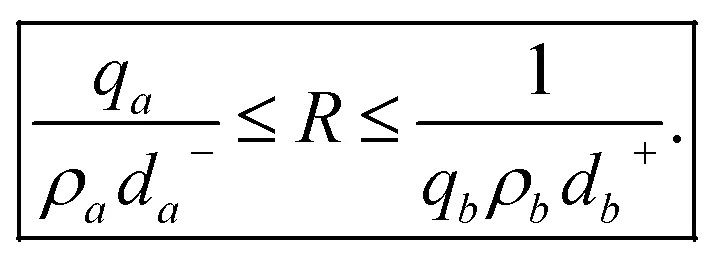


Every ratio in this interval guarantees both fidelity targets on a positive concentration ray. The interval is non-empty exactly when *q*_*a*_*q*_*b*_ ≤ *P*_*L*_. Occupancy and the declared bounded concentration domain still determine whether a usable point on that ray exists; the next section gives the exact answer to that bounded problem.

## 4 Complete fixed-one-total formulation window

Fix *G*_*i*,T_ > 0 and let *G* = *G*_*j*,T_ ∈ [*L*, *U*], where 0 < *L* ≤ *U* < *∞*. At either condition, *Φ*_*i*_ decreases strictly with *G*, *Φ*_*j*_ increases strictly, and occupancy is non-decreasing (SI Section 4).

Suppose fixed guest *i* occupies fraction *β* ∈ (0, 1) of occupied host. Eliminating *X*_*i*_ gives a quadratic in *h*. Define13
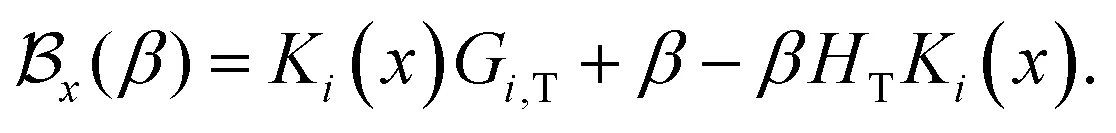


The physical root and corresponding competitor total are14
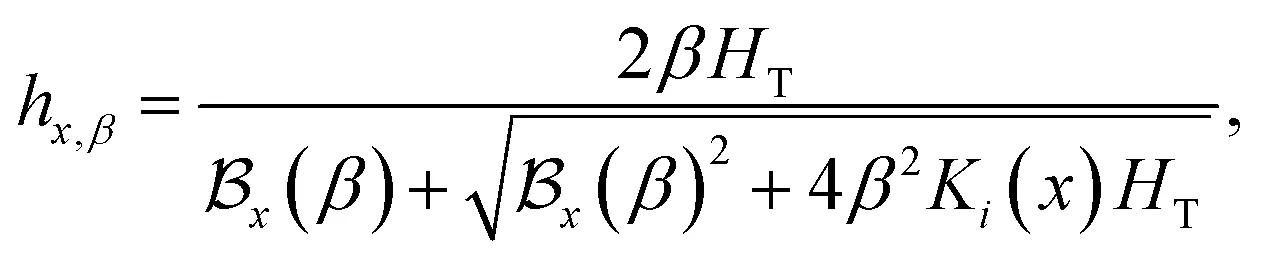
15
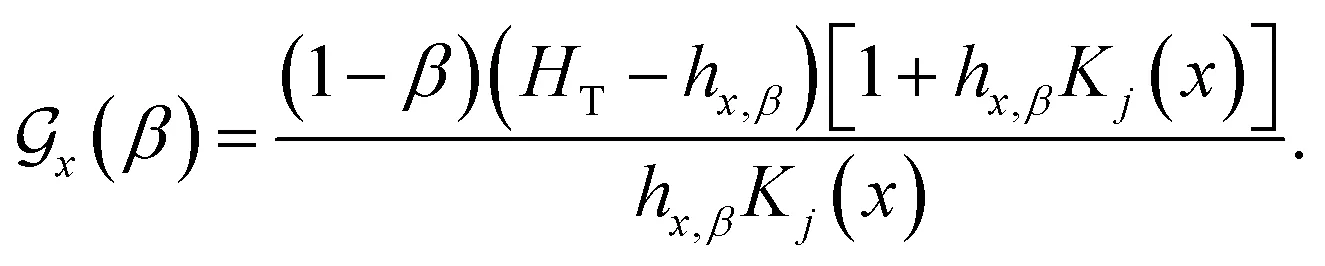
Closed-form occupancy thresholds 
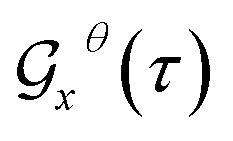
 are given in the SI.

Theorem 1 (bounded two-condition formulation interval). Let 0 < *η*_*a*_, *η*_*b*_ < 1 and 0 ≤ *τ*_*a*_, *τ*_*b*_ < 1. Define16
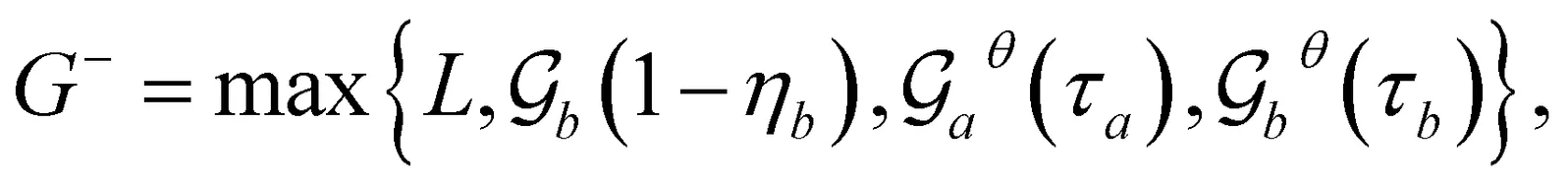
17
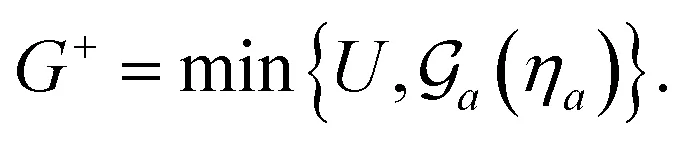
Within the ideal one-site two-guest model, the specifications *Φ*_*i*_(*a*) ≥ *η*_*a*_, *Φ*_*j*_(*b*) ≥ *η*_*b*_, *θ*(*a*) ≥ *τ*_*a*_ and *θ*(*b*) ≥ *τ*_*b*_ are jointly feasible if and only if *G*^−^ ≤ *G*^+^. If feasible, the complete admissible set is [*G*^−^, *G*^+^].

The theorem returns more than a yes/no answer. The active term in *G*^−^ or *G*^+^ identifies whether the limiting requirement is domain, first-condition fidelity, second-condition fidelity or occupancy.

Experimentally, [*L*, *U*] is the allowed addition range for guest *j*, for example a range set by solubility or sample preparation. The lower boundary *G*^−^ is the largest amount required to make *j* dominate at condition *b*, meet either occupancy floor, or satisfy the experimental lower limit. The upper boundary *G*^+^ prevents too much *j* from displacing target *i* at condition *a*, and also respects the experimental upper limit. Equality means that the active target is just met. An overlap gives all acceptable recipes; if *G*^−^ > *G*^+^, changing *j* alone cannot meet the specification. The restrictions *η* < 1 and *τ* < 1 exclude exact 100% performance at finite positive concentrations and finite affinities.

### 4.1 A hypothetical pH 12/2 formulation workflow

To illustrate an experimental design decision, consider guests A and B with the hypothetical, fixed effective affinities18
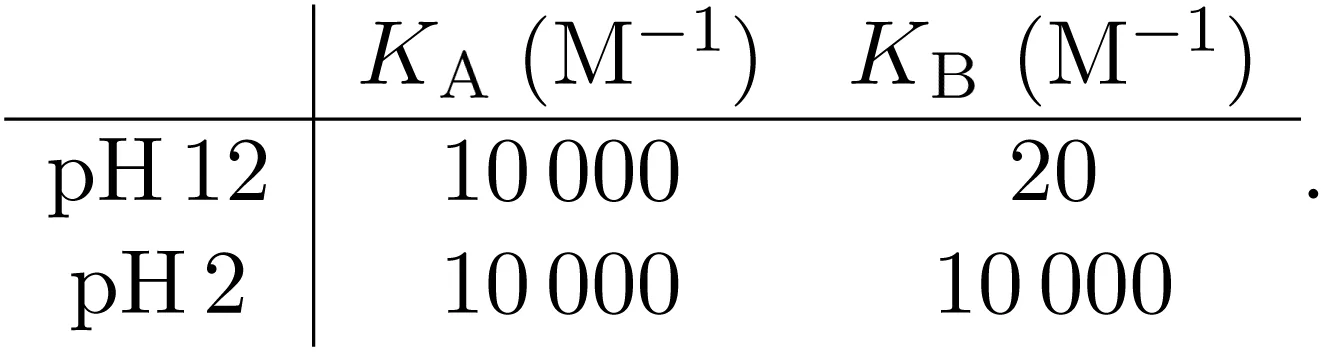


These hypothetical values do not represent either experimental case study. The example assumes that the same host and one-site binding model remain applicable at both pH values. Here “95% accuracy” means that at least 95% of occupied host contains A at pH 12 and B at pH 2; a separate occupancy floor of 50% is imposed at each condition. Fix [A]_T_ = 0.20 mM and allow [B]_T_ ∈ [0.001, 20] mM. At *H*_T_ = 0.20 mM, the pH 2 requirement gives [B]_T_ ≥ 3.800000 mM, whereas the pH 12 requirement gives [B]_T_ ≤ 2.682638 mM. The empty intersection diagnoses conflicting fidelity requirements: no adjustment of B alone can meet the specification at this fixed host total. The controlled-free reference is 95.7193%, but it does not describe this finite-total mixture.

Reducing only *H*_T_ to 0.050 mM and recalculating gives the complete interval19
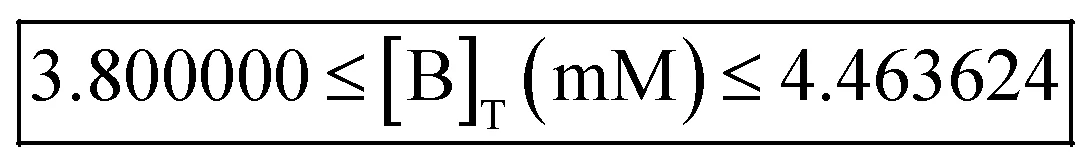


Both occupancy thresholds are zero, so the independently imposed 50% floors do not narrow this interval. A candidate recipe is therefore *H*_T_ = 0.050 mM, [A]_T_ = 0.20 mM and [B]_T_ = 4.000 mM at each equilibrated pH endpoint, accounting for any dilution during pH adjustment. Direct mass-balance calculations give *Φ*_A_(12) = 95.4935%, *Φ*_B_(2) = 95.2381%, *θ*(12) = 63.9586% and *θ*(2) = 97.6477%. The corresponding desired-complex yields are 61.0764% and 92.9978%, so 95% conditional fidelity is not a claim of 95% host conversion. Before laboratory use, the effective constants, equilibrium, host stability and applicability of the one-site model would have to be checked at both pH endpoints. The calculation specifies the endpoint compositions and does not predict a switching rate or stability under repeated pH cycling.

The analytical construction gives the limiting thresholds and their intersection directly. Using the same monotonicity result, bracketed numerical threshold searches recover the interval to numerical tolerance. Table 2 illustrates how a coarse grid can miss a feasible interval. Full threshold, grid and endpoint checks are provided in the SI.

**Table 2:** Direct comparison for the hypothetical formulation after reducing *H*_T_ to 0.050 mM. Numerical methods use the same finite-total equilibrium model and concentration domain. Concentrations are in mM

Method	Result for [B]_T_	Interpretation
Closed-form thresholds	[3.800000, 4.463624]	Complete interval from the proved monotonicity
Forward solver + root finding	[3.800000, 4.463624]	Same endpoints to numerical tolerance; monotonicity establishes completeness
8-Point uniform grid	No feasible sample	A coarse scan misses this interval
21-Point uniform grid	4.000800 only	A feasible sampled recipe; the sample alone does not locate both boundaries

## 5 Pairwise observation consistency with additional competitors

The exact formulation interval above is binary. A different statement remains valid when further guests compete for the same site.

Corollary 1 (pairwise consistency in an *N*-guest mixture). For one mutually exclusive 1 : 1 host site and any finite number of guests, the physical free-host concentration is unique. For any selected pair *i*, *j*,20
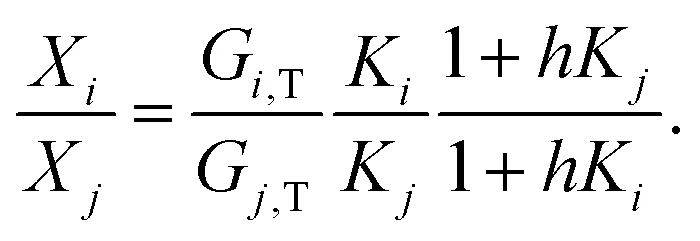


Consequently,21
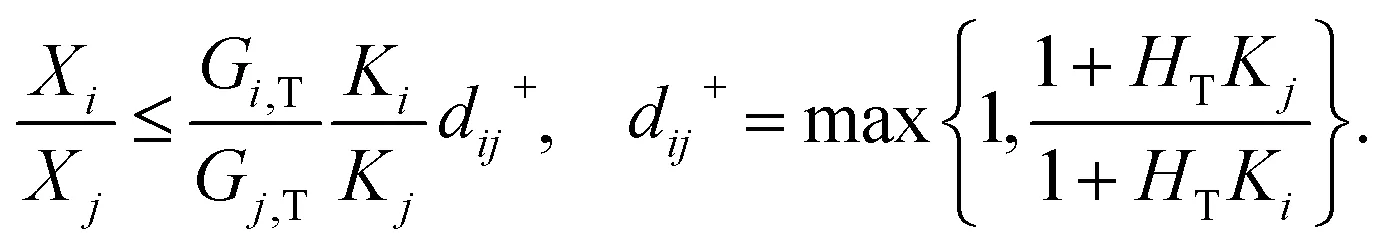


An experimental lower bound exceeding this endpoint value rejects the specified same-site parameterization. Additional competitors affect the pairwise ratio only through the common *h*. The corollary supplies a pairwise bound; complete multiguest formulation intervals would require additional derivation.

The proof is given in SI Section 2. This result allows the pairwise consistency analysis below to use a three-guest observation while keeping the binary inverse-design theorem separate from a full three-guest formulation.

## 6 Two-variable concentration-region construction

The fixed-one-total theorem gives a closed interval because every requirement becomes a one-sided constraint on a single variable. If two guest totals are allowed to vary independently, the same one-site mass balance still gives an exact boundary construction, but the admissible set is generally a two-dimensional region rather than an interval.

Let *u* = *G*_*p*,T_ and *v* = *G*_*q*,T_ be the two variable guest totals. At condition *x*, define22
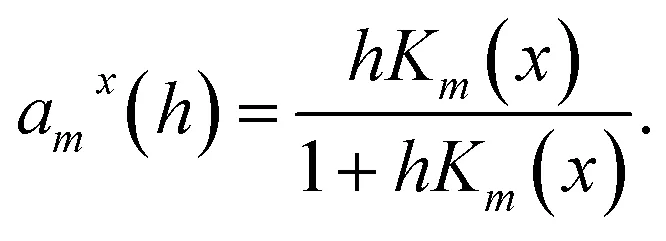


After all fixed guest contributions have been collected, the host balance has the linear form23
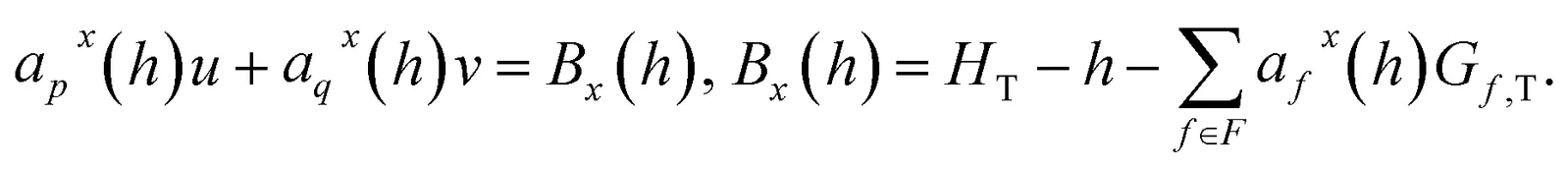


A fidelity equality for a target guest *t* at level *η* can also be written as a linear equation in (*u*, *v*) at fixed *h*:

24 *c*_*p*_^*x*^(*h*)*u* + *c*_*q*_^*x*^(*h*)*v* = *D*_*x*_(*h*;*η*,*t*).

If the target is one of the variable guests, for example *t* = *p*, then *c*_*p*_^*x*^ = (1 − *η*)*a*_*p*_^*x*^, *c*_*q*_^*x*^ = −*ηa*_*q*_^*x*^ and 
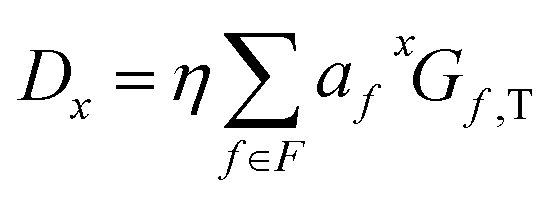
. Combining eqn (23) and (24) gives the parametric boundary25
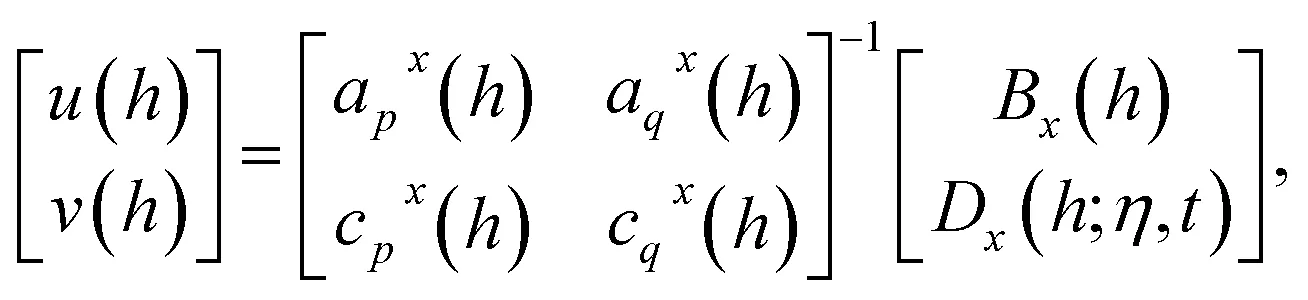
where the determinant is non-zero and both totals are positive. If instead *t* ∈ *F* is fixed, then *c*_*m*_^*x*^ = −*ηa*_*m*_^*x*^ and 
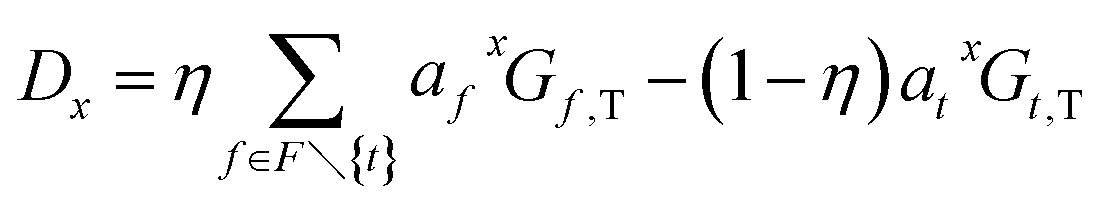
. The two matrix rows are proportional, so this case is obtained without inversion: first solve *a*_*t*_^*x*^(*h*)*G*_*t*,T_ = *η*(*H*_T_ − *h*) for *h*, then use eqn (23) to obtain a straight line in (*u*, *v*). Only positive points inside the declared concentration rectangle are retained. A fidelity boundary therefore marks recipes that just meet one target; the original inequality identifies the admissible side. For 0 < *τ* < 1, the occupancy requirement is the half-plane *a*_*p*_^*x*^(*h*_*τ*_)*u* + *a*_*q*_^*x*^(*h*_*τ*_)*v* ≥ *B*_*x*_(*h*_*τ*_), where *h*_*τ*_ = *H*_T_(1 − *τ*); at *τ* = 0 it is automatic. These boundary constructions do not imply that the final region is convex or described by one pair of endpoints.

## 7 Published coordination-cage application

### 7.1 System and transfer assumptions

Cullen and co-workers reported a Co_8_L_12_ cage that selects adamantane-1,3-dicarboxylic acid (H_2_A) at low pH, cyclononanone (C) at intermediate pH and 1-aminoadamantane (B) at high pH.^1,2^ The combined three-guest experiment contained 0.20 mM cage, 0.75 mM H_2_A, 0.20 mM C and 7.10 mM B, whereas the separate C/B pH-series experiment used 0.20 mM cage, 0.20 mM C and 2.0 mM B.^2^ The binding inputs used here are *K*_C_ = 1.1 × 10^4^ M^−1^, *K*^0^_B_= 1.0(3)×10^4^ M^−1^, *K*_BH^+^_ = 13(7) M^−1^ and p*K*_a_ = 10.9.^1,2^ D_2_O is the solvent used for the source ^1^H NMR binding and pH-titration measurements, rather than a solvent selected for the present calculation.^1,2^ A deuterated solvent provides the NMR deuterium lock and reduces the solvent proton background. The reported operational acidity values are retained when transferring the source parameters. A glass-electrode reading in D_2_O is distinct from pD and requires a stated conversion convention.^46^ Applying the same additive scale correction to both pH and p*K*_a_ leaves the difference p*K*_a_ − pH, and hence the fraction in eqn (26), unchanged. This bookkeeping does not rule out solvent isotope effects or binding-induced protonation shifts, which remain outside the transferred parameterization.^47,48^

The transferred effective B affinity is26
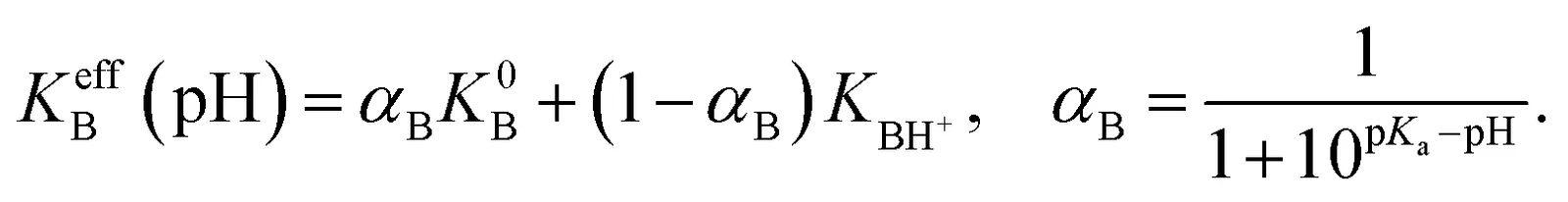


For the central values, *K*^eff^_B_(7.5) = 16.97 M^−1^ and *K*^eff^_B_(12.2) = 9523.35 M^−1^. Eqn (26) assumes rapid protonation pre-equilibrium, unchanged free-solution protonation fractions in the competitive matrix and quantitative transfer of the binary state-specific affinities. The application therefore uses a three-level interpretation:

(1) The inverse-design theorem is exact only within the ideal two-guest, one-site model;

(2) The application transfers binary constants and a free-solution protonation pre-equilibrium into the mixture;

(3) Any mismatch may reflect non-transferable constants, activity effects, pH/pD differences, external association, multiple sites, binding-induced protonation shifts or another omitted mechanism. The calculation identifies inconsistency while leaving the microscopic cause unresolved.

### 7.2 Pairwise consistency of the reported three-guest C/B observation

The strongest data-facing test uses the combined three-guest experiment rather than a chosen design threshold. At pH 7.5, the source reports more than 97% cage-C and less than 2% cage-B, implying *X*_C_/*X*_B_ > 48.5. Because the pairwise corollary remains valid with additional mutually exclusive cavity competitors, including H_2_A, the transferred same-site model requires

27 *K*^eff^_B_(7.5) < 6.38885 M^−1^.

At fixed reported p*K*_a_ = 10.9, the rectangular range formed from the quoted marginal uncertainties,

28 *K*^0^_B_ ∈ [7000,13 000] M^−1^, *K*_BH^+^_ ∈ [6,20] M^−1^,

has minimum *K*^eff^_B_(7.5) = 8.78325 M^−1^, already above the consistency boundary. Every point in this rectangle is therefore incompatible with the reported pairwise composition under the transferred same-site model. The statement has deterministic range-exclusion meaning; confidence-region and hypothesis-testing interpretations, as well as the complete three-guest population profile, lie outside its scope.

This exclusion can be read directly from the protonation balance. At pH 7.5 and p*K*_a_ = 10.9, the neutral fraction of B is 3.98 × 10^−4^, so B is 99.96% protonated. The neutral term is nevertheless not negligible because *K*^0^_B_ is large: over the quoted *K*^0^_B_ = 7000–13 000 M^−1^ range, *α*_B_*K*^0^_B_ contributes 2.79–5.17 M^−1^ to *K*^eff^_B_. Therefore even the favourable corner *K*^0^_B_ = 7000 M^−1^ and *K*_BH^+^_ = 6 M^−1^ gives *K*^eff^_B_(7.5) = 8.78325 M^−1^, above the consistency bound. At the fixed reported acidity convention, the favourable-corner value is about 37% above the allowed bound. The conditional sensitivity calculation requires p*K*_a_ > 11.755 at pH 7.5; it does not establish a physical p*K*_a_ shift or exclude solvent-dependent changes in the transferred parameters.

Fig. 1 separates two questions. Below the red boundary, some B total can satisfy the binary 95%/95% fidelity specification at fixed host and C totals. The blue boundary is a necessary screen for the reported three-guest C/B ratio: points above it are excluded, whereas points below it are not excluded by this bound alone. Passing that screen does not establish reproduction of the full mixture. The shaded regions distinguish these outcomes. The quoted-uncertainty rectangle partly enters the binary-feasible region but lies entirely above the necessary observation boundary.

**Fig. 1:**
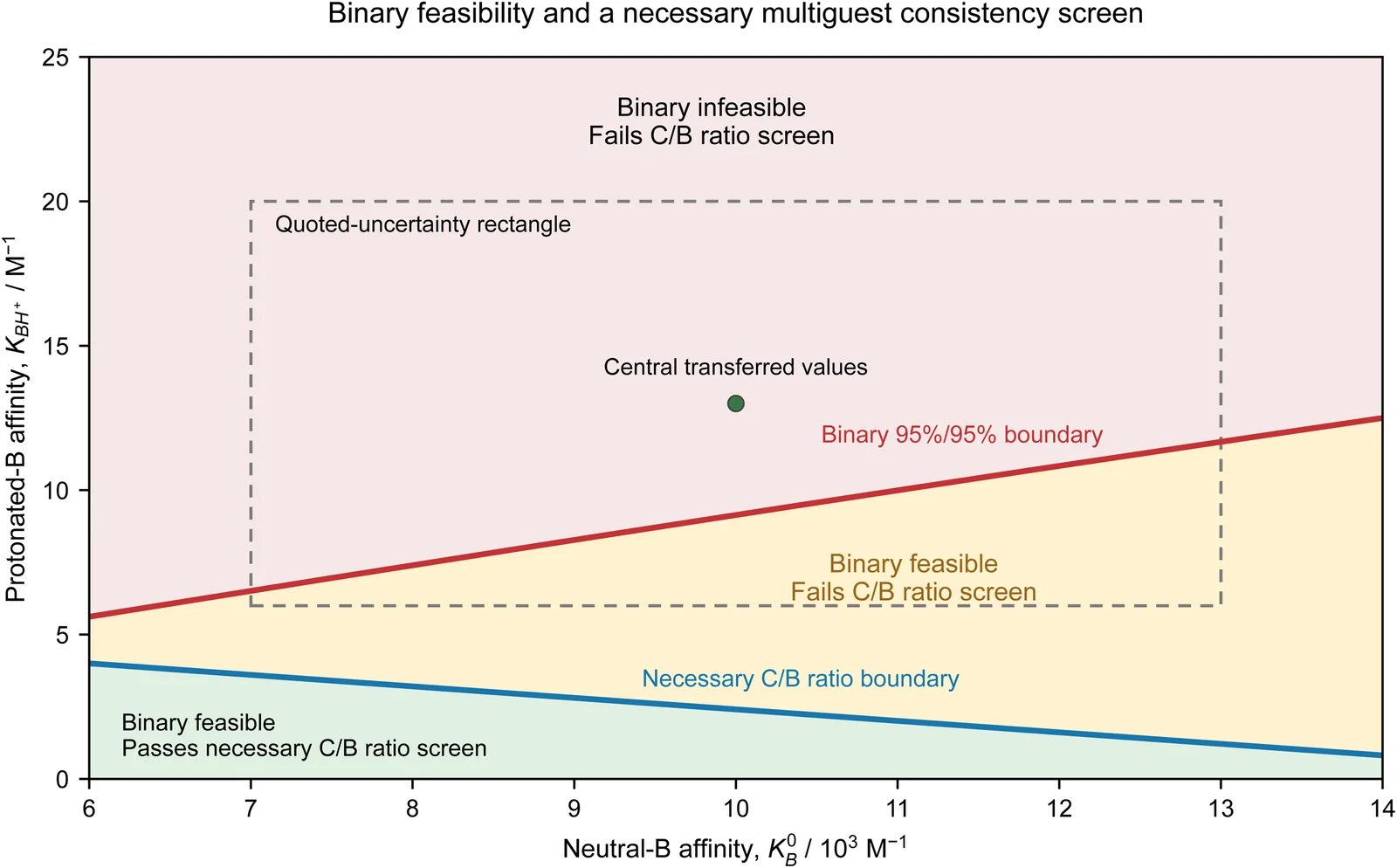
Two model-conditional Cullen calculations with *H*_T_ = [C]_T_ = 0.20 mM. The red curve marks a singleton binary 95%/95% formulation interval; points on or below it are feasible as [B]_T_ is varied. The blue line is the necessary pairwise observation bound at the reported three-guest totals: *K*^eff^_B_(7.5) < 6.38885 M^−1^. Shading below blue denotes binary feasibility and passage of this necessary screen; shading between blue and red denotes binary feasibility but failure of the observation screen; above red both tests fail. A point below blue is not proof of reconstructing the full mixture, and equality on blue does not satisfy the strict observed ratio. The quoted marginal-uncertainty rectangle partly overlaps binary feasibility but is entirely excluded by the observation screen.

A conditional p*K*_a_ sensitivity calculation is given in the SI. At the most favourable corner of the quoted ranges, observation consistency would require p*K*_a_ > 11.755, approximately 0.85 units above the reported value; the central *K*_BH^+^_ = 13 M^−1^ already exceeds the bound in eqn (27) for every protonation fraction. The source article also notes that non-bound A^2−^ alters the appearance of bound-C signals, consistent with association between the polycationic cage and free A^2−^.^2^ Such effects are outside the ideal transferred same-site model and reinforce the interpretation as model or parameter non-transferability.

Three features distinguish this exclusion from the general expectation that binary constants may transfer imperfectly to competitive mixtures: (i) the test uses only quantities reported in the source (the stated composition and the authors' own constants) and introduces no fitted or auxiliary parameter, so the exclusion concerns the internal consistency of the published dataset, (ii) it excludes not a point estimate but the entire quoted marginal-uncertainty region, with its most favourable point still giving *K*^eff^_B_(7.5) = 8.78 M^−1^ against a required 6.39 M^−1^, so no combination within the reported uncertainties is compatible, and (iii) the gap is quantified as a parameter displacement, where restoring consistency at that point requires p*K*_a_ > 11.755, about 0.85 units above the reported value. A qualitative expectation of imperfect transfer predicts neither a gap of this size nor that it will survive across the full stated uncertainty region.

### 7.3 Binary C/B concentration-window projection

The one-variable interval calculation answers a separate design question. H_2_A is omitted, [C]_T_ = 0.20 mM is fixed, [B]_T_ is varied, and the exact two-guest interval theorem is applied at pH 7.5 and 12.2. Its scope is limited to the two-guest C/B projection; the full three-guest calculation is considered separately in Section 7.4. Because the source performance statement is primarily a bound-state composition, the calculation sets the occupancy floors to zero; positive floors can be imposed with the same theorem. The controlled-free-concentration reference for the central transferred constants is 95.95%. Taken alone, that value would suggest that a symmetric 95% handoff should be attainable. The 94% and 95% thresholds are imposed design specifications used to illustrate the concentration-window calculation.

The binary finite-depletion calculation gives a different decision. The two condition-specific fidelities are equal at

29 [B]_T_ = 3.8371 mM, *Φ*_C_(7.5) = *Φ*_B_(12.2) = 94.36%.

This point is the closed-system maximin. The predicted host occupancies at this point are 53.56% at pH 7.5 and 97.36% at pH 12.2. A symmetric 94% requirement has the complete non-empty interval

30 3.5908 ≤ [B]_T_/mM ≤ 4.1042.

At 95%, however, the high-pH B requirement gives

31 [B]_T_ ≥ 4.3604 mM,

while the middle-pH C requirement gives

32 [B]_T_ ≤ 3.3710 mM.

Their non-overlap proves that concentration tuning alone cannot meet 95%/95% under the central transferred binary parameterization. At [B]_T_ = 7.10 mM, the B total used in the combined three-guest experiment, this binary projection predicts 90.19% C fidelity at pH 7.5 and 96.86% B fidelity at pH 12.2 (Fig. 2).

**Fig. 2:**
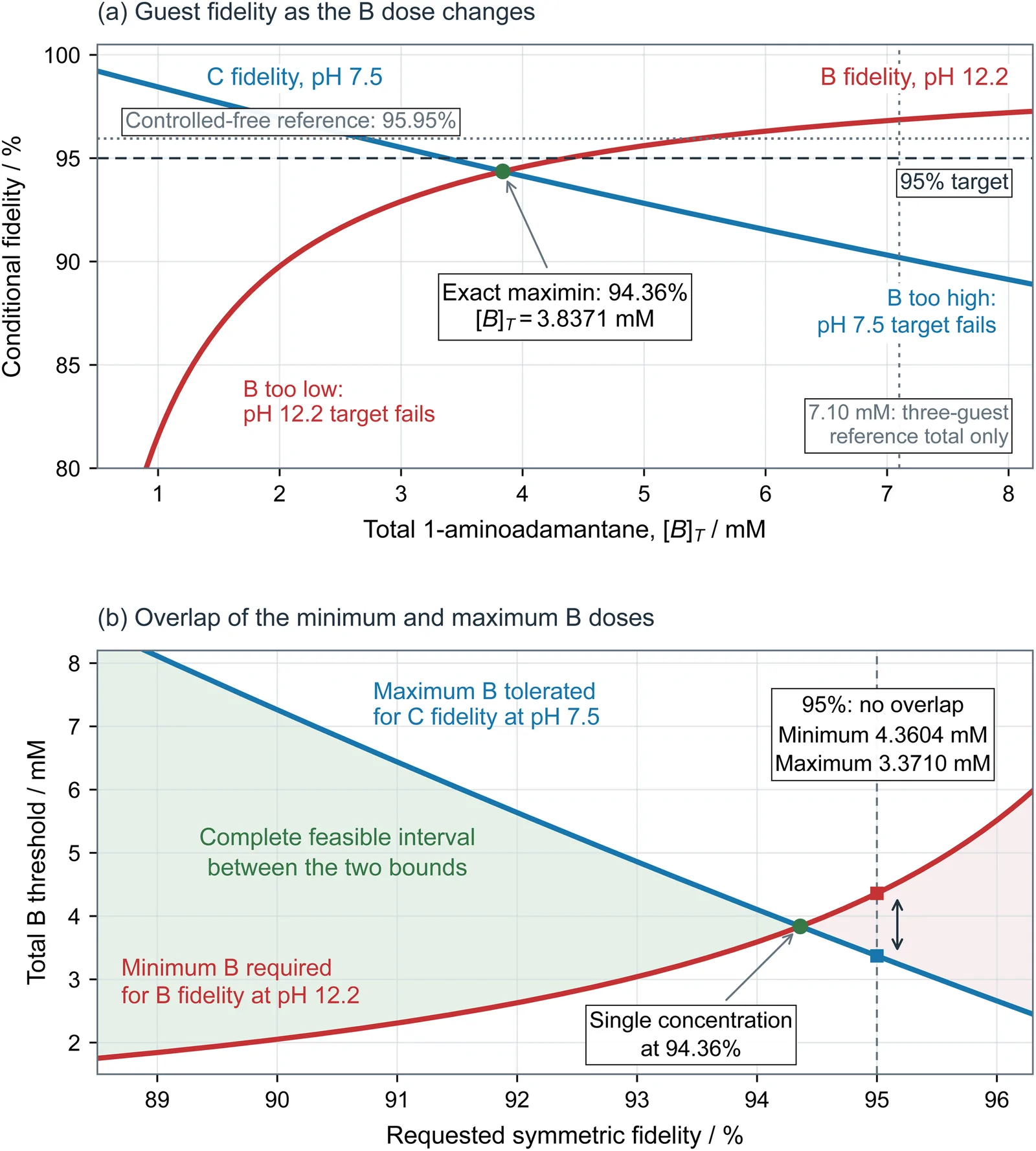
Binary C/B concentration-window projection using the central transferred affinities, with *H*_T_ = [C]_T_ = 0.20 mM and zero occupancy floors. (a) Adding B improves B fidelity at pH 12.2 but displaces too much C at pH 7.5. Their crossing gives the exact maximin of 94.36% at [B]_T_ = 3.8371 mM. At 95%, C requires [B]_T_ ≤ 3.3710 mM while B requires [B]_T_ ≥ 4.3604 mM, so no B total meets both. The 7.10 mM marker is only the B-total reference from the separate three-guest context. (b) The green overlap between the lower B requirement and upper C limit is the complete feasible interval. It contracts to a single concentration at the exact maximin of 94.36%, below both the 95% request and the 95.95% controlled-free reference. Colours identify the same guest constraint in both panels.

Part of the reported marginal parameter rectangle remains binary-feasible. At the favourable corner (*K*^0^_B_,*K*_BH^+^_) = (7000,6) M^−1^, the exact 95% interval is approximately 6.1498–6.5097 mM. This parameter sensitivity is distinct from the three-guest observation consistency analysis above.

### 7.4 Two-variable full three-guest Cullen region

The two-variable construction was applied to the full Cullen acid/C/B system by fixing [C]_T_ = 0.20 mM and varying both [H_2_A]_T_ and [B]_T_. Three pH conditions were considered: pH 3.0 with H_2_A as target, pH 7.5 with C as target and pH 12.2 with B as target. The neutral diacid affinity was taken as 2.3 × 10^5^ M^−1^, while the charged H_2_A affinity was treated in two ways: the reported upper bound *K*_A^2−^_ = 300 M^−1^ and a favourable zero-affinity limit. The latter is included because the charged affinity was reported only as an upper limit.^1,2^

Under the 300 M^−1^ upper-bound model, the best point found in the declared two-variable region gives a minimum three-state fidelity of 90.43% at [H_2_A]_T_ = 0.261 mM and [B]_T_ = 2.246 mM. In the favourable zero-charged-affinity limit, the corresponding maximum rises to 94.15% at [H_2_A]_T_ = 0.336 mM and [B]_T_ = 3.692 mM. The published totals, 0.75 mM H_2_A and 7.10 mM B, give approximately (98.2, 77.1, 96.5)% in the upper-bound model and (98.2, 89.6, 96.9)% in the zero-affinity limit for the low-, middle- and high-pH targets. Thus the two-variable map gives an independent route to the same conclusion as the pairwise consistency test: the transferred same-site constants underpredict the middle-pH dominance of C (Fig. 3).

**Fig. 3:**
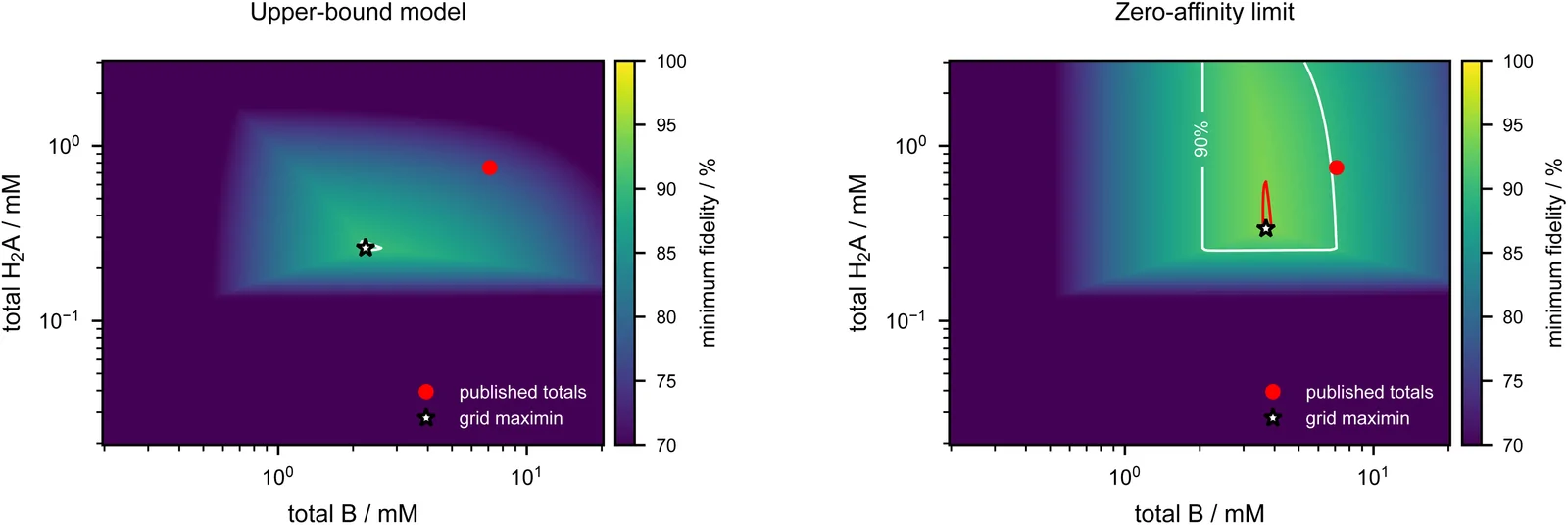
Two-variable full three-guest Cullen concentration maps. The colour scale is the minimum of the three target fidelities: H_2_A at pH 3.0, C at pH 7.5 and B at pH 12.2. Left: charged H_2_A affinity set to the reported upper bound of 300 M^−1^. Right: favourable zero-affinity limit for charged H_2_A. The published three-guest totals and the numerical maximin points are marked. These maps use the exact two-variable boundary construction for interpretation but do not claim a one-interval topology.

## 8 Second literature application: pH-responsive naphthotube switch

The same fixed-one-total calculation was applied to the pH-responsive naphthotube 5b/G4/G5 system reported by Wang and co-workers. That study gives the necessary two-condition affinity matrix for host 5b: G4 binds strongly at pH 7.4 (*K* = 1.7 × 10^4^ M^−1^) and weakly at pH 12 (*K* = 90 M^−1^), whereas G5 binds weakly at pH 7.4 (*K* = 20 M^−1^) and strongly at pH 12 (*K* = 5.9 × 10^3^ M^−1^).^45^ The source paper also reports reversible guest swapping in an equimolar ternary mixture of 5b, G4 and G5 upon acid/base addition.^45^

This second system marks the high-selectivity-margin regime. The intrinsic selectivity swing is *S* ≈ 5.6 × 10^4^, compared with approximately 5.6 × 10^2^ for the Cullen C/B projection. Using a scale of *H*_T_ = [G4]_T_ = 0.10 mM and varying total G5, the controlled-free-concentration reference is 99.58%. Exact finite-depletion analysis gives a closed-system maximin of 99.35% at [G5]_T_ = 0.292 mM, a penalty of only 0.22 percentage points (Fig. 4). A 95%/95% specification has a broad exact interval,

33 0.0432 ≤ [G5]_T_/mM ≤ 2.3969.

**Fig. 4:**
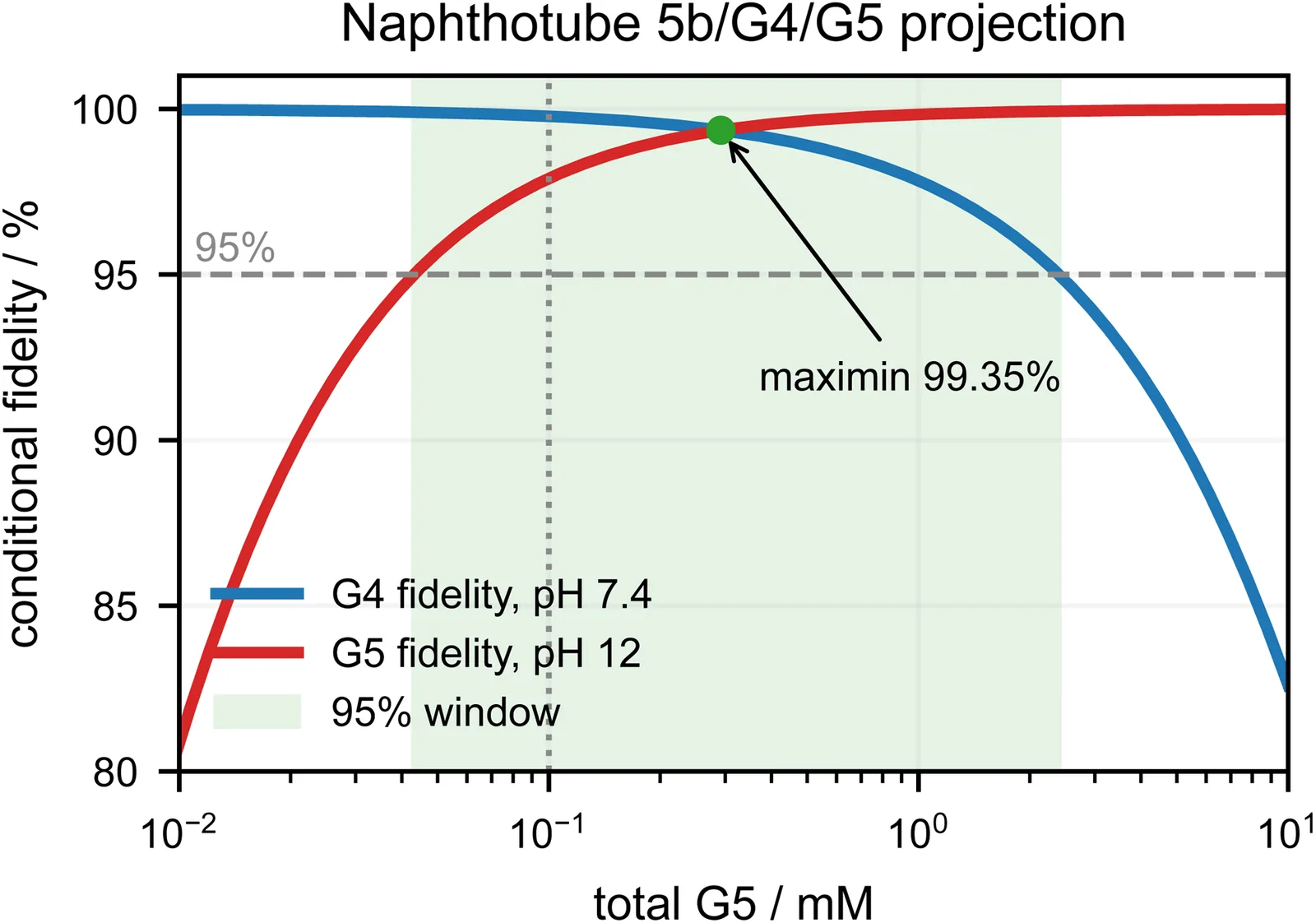
Second literature application using the pH-responsive naphthotube 5b/G4/G5 affinity matrix. With *H*_T_ = [G4]_T_ = 0.10 mM and variable total G5, the exact 95% concentration window spans 0.0432–2.3969 mM. The equimolar 0.10 mM point lies well inside this interval. Unlike the Cullen C/B projection, this high-selectivity-margin system is insensitive to the small finite-depletion penalty.

At an equimolar 0.10 mM G5 total, the predicted fidelities are 99.78% for G4 at pH 7.4 and 97.90% for G5 at pH 12.

The naphthotube comparison illustrates the contrasting limiting case: when the selectivity margin lies far above the requested threshold, finite depletion has little practical effect and the simple reference is adequate. Together with the Cullen case, this identifies where the finite-depletion calculation matters most—near the selectivity margin.

## 9 Discussion

The Cullen application illustrates a model-conditional consistency test. The pairwise bound excludes the quoted B-affinity rectangle under direct same-site transfer, and the full three-guest calculation also underpredicts middle-pH C dominance at the published totals. Both calculations use the same transferred assumptions; they do not independently validate the actual mixture mechanism. The reported experiment demonstrates pH-controlled selection, while the calculation identifies a limitation of this parameterization.

The two literature examples illustrate different distances from a requested fidelity threshold. In the Cullen binary projection the controlled-free reference is 95.95%, just above 95%, whereas the exact maximin is 94.36% and the 95% window is empty. For the naphthotube, the reference and exact values are 99.58% and 99.35%, respectively, so the 95% window remains broad. These examples show how depletion can change a decision near a specified threshold; they do not establish that it is negligible for every system with a large selectivity swing.

For one varied total in the binary model, monotonicity converts every fidelity and occupancy requirement into a one-sided constraint. The intersection provides both the complete admissible interval and its active limiting requirements. This helps separate a formulation that needs concentration adjustment from a parameterization that fails an observation-consistency test. The hypothetical workflow in Section 4.1 demonstrates the former: changing the host total opens a window that varying B alone cannot achieve.

The result complements parameter estimation and numerical speciation. Titration, calorimetry and global fitting provide binding inputs; general equilibrium solvers return populations for chosen totals and can support numerical inverse searches.^17,18,24,38,42^Table 2 shows that numerical equilibrium plus bracketed threshold finding agrees with the closed-form result. The analytical construction exposes the thresholds directly and supplies the monotonicity proof used to establish completeness. More complex binding models may require numerical methods and a separate analysis of their feasible sets.

The multiguest corollary preserves the pairwise observation-consistency bound in the presence of additional same-site competitors. The binary inverse-design theorem remains a two-guest formulation result, while general multiligand equilibrium solvers already address the forward problem.^38^ The two-variable construction supplies exact boundary curves and in-rectangle evaluation, but it does not imply convexity or a one-interval topology for arbitrary multiguest design spaces.

## 10 Conclusions

The established finite-depletion one-site model can be completed as a bounded inverse-design problem. Monotonicity converts independent fidelity and occupancy requirements into one-sided concentration constraints, and their intersection is the complete admissible formulation window for one varied guest total. The odds-product representation supplies a complementary global screen, while its pairwise endpoint form remains valid when additional guests compete for the same site. When two totals are varied independently, exact free-host-parametrized boundary curves replace the one-dimensional interval and support concentration-region mapping without a convexity claim.

The Cullen cage provides the main chemistry-facing test. The reported middle-pH three-guest C/B composition excludes the quoted B-affinity rectangle under direct same-site transfer, and the full acid/C/B two-variable map gives the same qualitative message by underpredicting C dominance at the published totals. The binary C/B projection then shows how finite depletion can flip a design decision near the selectivity boundary: a 95.95% controlled-free reference becomes a 94.36% exact closed-system maximum. The naphthotube comparison marks the other regime, where a much larger selectivity swing leaves a broad 95% concentration window and finite depletion is a minor correction. The overall result is a transparent model-conditional concentration-window and consistency analysis.

## Author contributions

Hyun Seo: conceptualization, methodology, formal analysis, software, validation, visualization, writing – original draft, writing – review & editing.

## Conflicts of interest

There are no conflicts to declare.

## Supplementary Material

RA-OLF-D6RA06172A-s001

RA-OLF-D6RA06172A-s002

RA-OLF-D6RA06172A-s003

## Data Availability

No new experimental measurements or source-figure digitization was performed. Published inputs and DOI-level provenance are listed in the article and supplementary information (SI). Mathematical derivations, proofs, numerical verification summaries, and additional tables are provided in the SI. The numerical data for the manuscript figures are provided in the supplementary workbook finite_depletion_inverse_design.xlsx. The analysis script run_analysis.py is supplied as a supplementary computational file. The additional script revision_analysis.py and its CSV/JSON outputs provide the hypothetical design example and independent numerical comparison. Supplementary information is available. See DOI: https://doi.org/10.1039/d6ra06172a.

## References

[cit1] Cullen W., Turega S., Hunter C. A., Ward M. D. (2015). Chem. Sci..

[cit2] Cullen W., Thomas K. A., Hunter C. A., Ward M. D. (2015). Chem. Sci..

[cit3] Pedersen C. J. (1967). J. Am. Chem. Soc..

[cit4] Cram D. J. (1983). Science.

[cit5] Rebek Jr J. (1987). Science.

[cit6] Lehn J.-M. (1988). Angew. Chem., Int. Ed. Engl..

[cit7] Schneider H.-J., Yatsimirsky A. K. (2008). Chem. Soc. Rev..

[cit8] Fujita M., Oguro D., Miyazawa M., Oka H., Yamaguchi K., Ogura K. (1995). Nature.

[cit9] Ward M. D., Hunter C. A., Williams N. H. (2018). Acc. Chem. Res..

[cit10] Kilbas B., Mirtschin S., Scopelliti R., Severin K. (2012). Chem. Sci..

[cit11] Han M., Michel R., He B., Chen Y.-S., Stalke D., John M., Clever G. H. (2013). Angew. Chem., Int. Ed..

[cit12] Zhang D., Ronson T. K., Güryel S., Thoburn J. D., Wales D. J., Nitschke J. R. (2019). J. Am. Chem. Soc..

[cit13] Ghosh A., Slappendel L., Nguyen B.-N. T., von Krbek L. K. S., Ronson T. K., Castilla A. M., Nitschke J. R. (2023). J. Am. Chem. Soc..

[cit14] Smulders M. M. J., Zarra S., Nitschke J. R. (2013). J. Am. Chem. Soc..

[cit15] Wiseman T., Williston S., Brandts J. F., Lin L.-N. (1989). Anal. Biochem..

[cit16] Turnbull W. B., Daranas A. H. (2003). J. Am. Chem. Soc..

[cit17] Thordarson P. (2011). Chem. Soc. Rev..

[cit18] Hibbert D. B., Thordarson P. (2016). Chem. Commun..

[cit19] Ulatowski F., Dąbrowa K., Bałakier T., Jurczak J. (2016). J. Org. Chem..

[cit20] Pollard T. D. (2010). Mol. Biol. Cell.

[cit21] Jarmoskaite I., AlSadhan I., Vaidyanathan P. P., Herschlag D. (2020). eLife.

[cit22] Morel F. M. M., Morgan J. J. (1972). Environ. Sci. Technol..

[cit23] Gampp H., Maeder M., Meyer C. J., Zuberbühler A. D. (1985). Talanta.

[cit24] Gans P., Sabatini A., Vacca A. (1996). Talanta.

[cit25] Alderighi L., Gans P., Ienco A., Peters D., Sabatini A., Vacca A. (1999). Coord. Chem. Rev..

[cit26] Morrison J. F. (1969). Biochim. Biophys. Acta.

[cit27] Henderson P. J. F. (1972). Biochem. J..

[cit28] Chang K.-J., Jacobs S., Cuatrecasas P. (1975). Biochim. Biophys. Acta.

[cit29] Wang Z.-X. (1995). FEBS Lett..

[cit30] Zhen J., Antonio T., Dutta A. K., Reith M. E. A. (2010). J. Neurosci. Methods.

[cit31] Cheng Y.-C., Prusoff W. H. (1973). Biochem. Pharmacol..

[cit32] De Lean A., Munson P. J., Rodbard D. (1978). Am. J. Physiol..

[cit33] Motulsky H. J., Mahan L. C. (1984). Mol. Pharmacol..

[cit34] Munson P. J., Rodbard D. (1988). J. Recept. Res..

[cit35] Kuzmič P. (2011). Anal. Biochem..

[cit36] Hulme E. C., Trevethick M. A. (2010). Br. J. Pharmacol..

[cit37] Becskereki G., Horvai G., Tóth B. (2021). Int. J. Mol. Sci..

[cit38] Blay V., Otero-Muras I., Annis D. A. (2020). Anal. Chem..

[cit39] Langmuir I. (1918). J. Am. Chem. Soc..

[cit40] Markham E. C., Benton A. F. (1931). J. Am. Chem. Soc..

[cit41] Myers A. L., Prausnitz J. M. (1965). AIChE J..

[cit42] Kaiblinger N., Hahn R., Beck J., Wang Y., Carta G. (2024). Adsorption.

[cit43] Rony P. R. (1968). Sep. Sci..

[cit44] Sirkar K. K. (1977). Sep. Sci..

[cit45] Wang X., Quan M., Yao H., Pang X.-Y., Ke H., Jiang W. (2022). Nat. Commun..

[cit46] Glasoe P. K., Long F. A. (1960). J. Phys. Chem..

[cit47] Baker B. M., Murphy K. P. (1996). Biophys. J..

[cit48] Onufriev A. V., Alexov E. (2013). Q. Rev. Biophys..

