# Supplementary material for: Exact concentration regions for stimulus-switched competitive binding under finite depletion
Source: RSC Adv. 2026 Oct 7;16(46):60814–24. doi: 10.1039/d6ra06172a (PMC13644731; doi:10.1039/d6ra06172a)
Supplement: Supplementary file 1 [file RA-016-D6RA06172A-s003.pdf]

Supplementary Information (SI) for RSC Advances.
This journal is © The Royal Society of Chemistry 2026

Supporting Information

Exact concentration regions for stimulus-switched competitive binding under finite depletion

Hyun Seo[a]
[a]Independent Researcher, Seoul, Republic of Korea. E-mail: leviseo2019@gmail.com
ORCID: 0009-0005-0524-4113

1 Notation, dimensions and model convention

The analysis uses an ideal concentration-based equilibrium model. Association constants have units of concentration^{-1}, all host and guest variables are molar concentrations unless a dimensionless benchmark is stated, and activity coefficients are set to unity. The phrase *controlled-free-concentration reference* denotes a hypothetical system in which the free guest ratio $r = [i]_{\text{free}}/[j]_{\text{free}}$ is externally controlled and avoids an activity interpretation.

The core assumptions are:

1. one host site;
2. mutually exclusive 1:1 complexes;
3. positive fixed condition-specific association constants;
4. ideal concentrations and no additional mass-balance species;
5. equilibrium at each condition.

All claims of exactness, necessity, sufficiency or impossibility are conditional on this model.

For two guests i, j at condition x,

$$X_m = \frac{hK_m G_{m,T}}{1 + hK_m}, \qquad H_T = h + X_i + X_j. \tag{1}$$

Conditional fidelity, occupancy and desired-complex yield are

$$\Phi_i = \frac{X_i}{X_i + X_j}, \qquad \theta = \frac{X_i + X_j}{H_T}, \qquad Y_i = \theta \Phi_i. \tag{2}$$

Table 1: Symbols used in the manuscript and Supporting Information. M denotes mol L^{-1}; 1 denotes a dimensionless quantity. Concentration units must be used consistently with the inverse-concentration units of the association constants. The explicitly dimensionless benchmark uses scaled quantities.

Symbol	Units	Meaning and practical interpretation
Conditions, concentrations and affinities		
$x \in \{a, b\}$; i, j, m, t	—	Condition label; guest labels, with t denoting the desired guest. In the binary design, i is targeted at a and j at b.
N; F; $f \in F$	—	Number of competing guests; set of guests whose totals are fixed; a member of that set. The set F differs from the balance function $F(h)$.
H_T; h, h_x	M	Total host concentration; free (unoccupied) host concentration at the condition considered.

Continued on next page

Table 1 continued

Symbol	Units	Meaning and practical interpretation
$G_{m,T}$; $[m]_T$	M	Total concentration of guest m, including its free and host-bound forms in the stated model.
$G = G_{j,T}$; $u = G_{p,T}$, $v = G_{q,T}$	M	Guest total varied in the one-variable design; the two independently varied totals in the concentration-plane construction.
$[m]_{\text{free}}$; $X_m(x)$	M	Free guest concentration; concentration of the host–guest complex containing m.
$K_m(x)$	M^{-1}	Association constant of guest m at condition x; larger values favour binding at a given free-guest concentration.
K_B^0, K_{BH^+}, K_B^{eff}	M^{-1}	Neutral-B, protonated-B and protonation-weighted effective B affinities in the Cullen application.
$K_{A^{2-}}$; K_{G4}, K_{G5}	M^{-1}	Charged-acid affinity in the Cullen model; affinities of the two named naphthotube guests.
α_B; pH, pK_a	1	Free-solution neutral fraction of B; acidity reading and dissociation parameter used to calculate that fraction under the stated convention.
Performance measures and two-condition selectivity		
Φ_t	1	Conditional fidelity: fraction of occupied host carrying the target guest, $X_t/\sum_m X_m$.
θ; $Y_t = \theta\Phi_t$	1	Fraction of all host that is occupied; fraction of all host carrying the target. High fidelity alone does not guarantee high yield.
O; O_a, O_b; $Q(G)$	1	Fidelity odds $\Phi/(1-\Phi)$; target odds at the two conditions; fixed-guest/variable-guest odds used in the monotonicity proof.
r; r_0; R	1	Free-guest ratio $[i]_{\text{free}}/[j]_{\text{free}}$; its fidelity-equalizing reference value; total-guest ratio $G_{i,T}/G_{j,T}$. Depletion generally makes r differ from R.
ρ_x; $S = \rho_a/\rho_b$	1	Affinity ratio $K_i(x)/K_j(x)$ at one condition; change in that ratio between the two conditions.
$d_x(h)$; $d_{ij}(h)$	1	Depletion correction $(1+hK_j)/(1+hK_i)$ connecting total-guest ratios to complex ratios.
d_x^-, d_x^+; d_{ij}^+	1	Lower and upper endpoint bounds on the depletion correction; the upper bound for a selected guest pair.
Δ; P_L, P_U	1	Ratio $d_a(h_a)/d_b(h_b)$ of depletion corrections; lower and upper bounds on the two-target odds product O_aO_b.
Requested specifications and concentration-window limits		
η, η_a, η_b; β	1	Requested target fidelity (for example, 0.95); fixed-guest fidelity used to calculate a threshold. At b, $\beta = 1-\eta_b$.
$q_x = \eta_x/(1-\eta_x)$	1	Requested fidelity expressed as target/non-target odds; 95% fidelity requires odds of 19.
τ, τ_a, τ_b	1	Minimum acceptable host occupancy, specified independently of fidelity.
L, U	M	Lowest and highest allowed competitor totals, defining the experimentally available formulation range.
G^-, G^+	M	Largest required lower bound and smallest allowed upper bound. Their overlap is the complete admissible concentration interval.
$\mathcal{G}_x(\beta)$	M	Competitor total at which the fixed guest has fidelity β.
$\mathcal{G}_x^\theta(\tau)$	M	Minimum competitor total needed to achieve occupancy τ at condition x.
$h_{x,\beta}$; h_τ	M	Free-host concentration at the fidelity threshold; free-host concentration $H_T(1-\tau)$ at the occupancy threshold.
$\mathcal{B}_x(\beta)$	1	Coefficient $K_i(x)G_{i,T} + \beta - \beta H_T K_i(x)$ in the fidelity-threshold quadratic; distinct from $B_x(h)$ below.

Continued on next page

Table 1 continued

Symbol	Units	Meaning and practical interpretation
Balance functions and two-variable boundaries		
$F(h)$; $F(h, G)$	M	Total host predicted from free and bound species minus the supplied host total. Its zero enforces host conservation.
$F'(h)$; F_h	1	Derivative of the host-balance function with respect to free host, with guest totals held fixed. Its positivity ensures a unique physical equilibrium.
$a_m^x(h)$	1	Fraction $hK_m(x)/(1 + hK_m(x))$ of guest m bound at a specified free-host concentration; $X_m = a_m^x G_{m,T}$.
$B_x(h)$	M	Occupied-host concentration that the two variable guests must supply after fixed-guest contributions have been subtracted.
$c_p^x(h), c_q^x(h)$; $D_x(h; \eta, t)$	1; M	Coefficients of the variable totals and the fixed-term right-hand side in the target-fidelity equality. They locate where a requested fidelity is just met.

2 Unique physical root and the multiguest pairwise corollary

For N mutually exclusive 1:1 guests,

$$X_m = \frac{hK_m G_{m,T}}{1 + hK_m}, \qquad F(h) = h + \sum_{m=1}^{N} \frac{hK_m G_{m,T}}{1 + hK_m} - H_T. \tag{3}$$

Because

$$F'(h) = 1 + \sum_{m=1}^{N} \frac{K_m G_{m,T}}{(1 + hK_m)^2} > 0, \tag{4}$$

F is strictly increasing. Since $F(0) = -H_T < 0$ and $F(H_T) > 0$, there is exactly one physical root in $(0, H_T)$.

For any pair i, j,

$$\frac{X_i}{X_j} = \frac{G_{i,T}}{G_{j,T}} \frac{K_i}{K_j} \frac{1 + hK_j}{1 + hK_i}. \tag{5}$$

The endpoint correction factor is bounded by

$$d_{ij}(h) = \frac{1 + hK_j}{1 + hK_i} \le d_{ij}^{+} = \max\left\{1, \frac{1 + H_T K_j}{1 + H_T K_i}\right\}. \tag{6}$$

Therefore

$$\boxed{\frac{X_i}{X_j} \le \frac{G_{i,T}}{G_{j,T}} \frac{K_i}{K_j} d_{ij}^{+}.} \tag{7}$$

Additional guests influence the pairwise ratio only through the common physical root h. This proves the multiguest observation-consistency corollary used in the Cullen consistency analysis. Complete multiguest inverse-design intervals would require additional derivation.

3 Two-guest odds factorization and asymmetric global bounds

Let guest i be targeted at condition a and guest j at condition b. Define

$$\rho_a = \frac{K_i(a)}{K_j(a)}, \qquad \rho_b = \frac{K_i(b)}{K_j(b)}, \qquad S = \frac{\rho_a}{\rho_b}, \tag{8}$$

$$d_x(h) = \frac{1 + hK_j(x)}{1 + hK_i(x)}, \qquad R = \frac{G_{i,T}}{G_{j,T}}. \tag{9}$$

The two target odds are

$$O_a = \frac{X_i(a)}{X_j(a)} = R\rho_a d_a(h_a), \tag{10}$$

$$O_b = \frac{X_j(b)}{X_i(b)} = \frac{1}{R\rho_b d_b(h_b)}, \tag{11}$$

which gives

$$\boxed{O_a O_b = S\Delta}, \qquad \Delta = \frac{d_a(h_a)}{d_b(h_b)}. \tag{12}$$

Since

$$\frac{\mathrm{d}d_x}{\mathrm{d}h} = \frac{K_j(x) - K_i(x)}{[1 + hK_i(x)]^2}, \tag{13}$$

d_x is monotone. Define

$$d_x^- = \min\{1, d_x(H_T)\}, \qquad d_x^+ = \max\{1, d_x(H_T)\}. \tag{14}$$

Then

$$P_L = S\frac{d_a^-}{d_b^+} \le O_a O_b \le S\frac{d_a^+}{d_b^-} = P_U. \tag{15}$$

For independent targets $0 < \eta_a, \eta_b < 1$, let

$$q_a = \frac{\eta_a}{1 - \eta_a}, \qquad q_b = \frac{\eta_b}{1 - \eta_b}. \tag{16}$$

If $P_U < q_a q_b$, simultaneous attainment is impossible within the ideal model.

A scale-free sufficient interval of total ratios follows from

$$O_a \ge R\rho_a d_a^-, \qquad O_b \ge \frac{1}{R\rho_b d_b^+}. \tag{17}$$

Therefore every ratio in

$$\boxed{\frac{q_a}{\rho_a d_a^-} \le R \le \frac{1}{q_b \rho_b d_b^+}} \tag{18}$$

guarantees both fidelity targets. It is non-empty exactly when $q_a q_b \le P_L$. The guarantee is limited to fidelity; occupancy and membership in a bounded concentration domain remain additional requirements.

4 Monotonicity for the fixed-one-total problem

Fix $G_{i,T}$ and write $G = G_{j,T}$. Suppress the condition label. The host balance is

$$F(h, G) = h + \frac{hK_i G_{i,T}}{1 + hK_i} + \frac{hK_j G}{1 + hK_j} - H_T = 0. \tag{19}$$

Implicit differentiation gives

$$\frac{\mathrm{d}h}{\mathrm{d}G} = -\frac{hK_j/(1 + hK_j)}{F_h}, \tag{20}$$

where

$$F_h = 1 + \frac{K_i G_{i,T}}{(1 + hK_i)^2} + \frac{K_j G}{(1 + hK_j)^2} > 0. \tag{21}$$

Hence h is non-increasing and occupancy $1 - h/H_T$ is non-decreasing.

The odds of fixed guest i over variable guest j are

$$Q(G) = \frac{G_{i,T} K_i}{G K_j} \frac{1 + hK_j}{1 + hK_i}. \tag{22}$$

Differentiation gives

$$\frac{\mathrm{d}\ln Q}{\mathrm{d}\ln G} = -1 + G\frac{\mathrm{d}h}{\mathrm{d}G}\left[\frac{K_j}{1+hK_j} - \frac{K_i}{1+hK_i}\right]. \tag{23}$$

If $K_j \geq K_i$, the correction in Eq. 23 is non-positive. If $K_i > K_j$, its positive magnitude is

$$\frac{GhK_j(K_i - K_j)}{(1+hK_j)^2(1+hK_i)F_h} < \frac{h(K_i - K_j)}{1+hK_i} < 1. \tag{24}$$

Therefore

$$\boxed{\frac{\mathrm{d}\ln Q}{\mathrm{d}\ln G} < 0} \tag{25}$$

for both affinity orders. Thus $\Phi_i(a; G)$ decreases strictly, $\Phi_j(b; G)$ increases strictly, and both occupancies are non-decreasing.

5 Closed-form asymmetric fidelity and occupancy thresholds

Suppose fixed guest i occupies fraction $\beta \in (0, 1)$ of occupied host:

$$X_i = \beta(H_T - h) = \frac{hK_iG_{i,T}}{1+hK_i}. \tag{26}$$

This gives

$$\beta K_i h^2 + (K_iG_{i,T} + \beta - \beta H_T K_i)\,h - \beta H_T = 0. \tag{27}$$

Define

$$\mathcal{B}_x(\beta) = K_i(x)G_{i,T} + \beta - \beta H_T K_i(x). \tag{28}$$

The stable positive root is

$$h_{x,\beta} = \frac{2\beta H_T}{\mathcal{B}_x(\beta) + \sqrt{\mathcal{B}_x(\beta)^2 + 4\beta^2 K_i(x) H_T}}. \tag{29}$$

The corresponding competitor total is

$$\boxed{\mathcal{G}_x(\beta) = \frac{(1-\beta)(H_T - h_{x,\beta})[1 + h_{x,\beta}K_j(x)]}{h_{x,\beta}K_j(x)}}. \tag{30}$$

Hence

$$\Phi_i(a) \geq \eta_a \iff G \leq \mathcal{G}_a(\eta_a), \tag{31}$$

$$\Phi_j(b) \geq \eta_b \iff G \geq \mathcal{G}_b(1-\eta_b). \tag{32}$$

For an occupancy floor $0 \leq \tau < 1$, set $h_\tau = H_T(1-\tau)$. The minimum competitor total is

$$\mathcal{G}_x^\theta(\tau) = \max\left\{0, \frac{\left[H_T - h_\tau - \dfrac{h_\tau K_i(x)G_{i,T}}{1+h_\tau K_i(x)}\right][1 + h_\tau K_j(x)]}{h_\tau K_j(x)}\right\}. \tag{33}$$

At $\tau = 0$, this is zero. Positive affinities and $\tau < 1$ ensure a finite threshold.

Theorem 1 (Asymmetric bounded formulation interval)**.** *Fix $G_{i,T} > 0$ and $G = G_{j,T} \in [L, U]$, $0 < L \leq U < \infty$. Let $0 < \eta_a, \eta_b < 1$ and $0 \leq \tau_a, \tau_b < 1$. Define*

$$G^- = \max\{L, \mathcal{G}_b(1-\eta_b), \mathcal{G}_a^\theta(\tau_a), \mathcal{G}_b^\theta(\tau_b)\}, \tag{34}$$

$$G^+ = \min\{U, \mathcal{G}_a(\eta_a)\}. \tag{35}$$

The full specification is feasible if and only if $G^- \leq G^+$. If feasible, the complete admissible set is $[G^-, G^+]$.

Proof. The monotonicity result converts the four specifications into one upper and three lower half-lines. Their intersection with $[L, U]$ is exactly $[G^-, G^+]$. □

6 Dimensionless benchmark

The benchmark uses $H_T = 1$, $G_{i,T} = 0.30$, $G \in [10^{-4}, 100]$, and

$$\{K_i(a), K_j(a), K_i(b), K_j(b)\} = \{3, 0.1, 30, 3\}. \tag{36}$$

For $\eta_a = \eta_b = 0.66$ and $\tau_a = \tau_b = 0.30$, the interval is

$$1.4793 \leq G \leq 1.6101. \tag{37}$$

The active lower constraint is $\mathcal{G}_a^\theta(0.30)$ and the active upper constraint is $\mathcal{G}_a(0.66)$; the occupancy floor at condition a therefore binds before the first-condition fidelity crossing. The unconstrained maximin of $\min\{\Phi_i(a), \Phi_j(b)\}$ occurs at the fidelity-equalizing point $G = 1.4623$, where $\Phi_i(a) = \Phi_j(b) = 67.98\%$. This point lies below $G^- = \mathcal{G}_a^\theta(0.30) = 1.4793$ and is excluded by the occupancy requirement, so the maximin over the admissible interval is attained at the endpoint G^-, where the binding metric is $\Phi_i(a) = 67.74\%$. The controlled-free-concentration reference maximin is 63.4%, while the exact bounded maximin is thus 67.7%, attained at G^- rather than at the interior crossing. The supplementary workbook contains the corresponding numerical grid.

7 Cullen input provenance and binary interval

The published inputs and source-experiment contexts are listed in Table 2. The binary interval calculation fixes $[C]_T = 0.20$ mM and varies $[B]_T$; the 7.10 mM B value is the B total from the combined three-guest experiment and is used as a reference concentration only. No source figure is digitized.

Table 2: Published inputs and source contexts used in the Cullen calculations.

Quantity	Value	DOI
H_T	0.20 mM	10.1039/C5SC01475A
$[C]_T$	0.20 mM	10.1039/C5SC01475A
Combined $[H_2A]_T$	0.75 mM	10.1039/C5SC01475A
Combined $[B]_T$ reference	7.10 mM	10.1039/C5SC01475A
Separate C/B $[B]_T$	2.0 mM	10.1039/C5SC01475A
K_C	1.10×10^4 M^{-1}	10.1039/C5SC01475A
K_B^0	$1.0(3) \times 10^4$ M^{-1}	10.1039/C4SC02090A
K_{BH^+}	13(7) M^{-1}	10.1039/C4SC02090A
$pK_a(BH^+)$	10.9	10.1039/C4SC02090A
Target pH values	7.5, 12.2	10.1039/C5SC01475A

The neutral fraction of B is

$$\alpha_B = \frac{1}{1 + 10^{pK_a - \mathrm{pH}}}, \tag{38}$$

and the transferred effective affinity is

$$K_B^{\mathrm{eff}} = \alpha_B K_B^0 + (1 - \alpha_B) K_{BH^+}. \tag{39}$$

For the central transferred values, the controlled-free-concentration reference is 95.95%, while the exact finite-depletion maximin is 94.36% at $[B]_T = 3.8371$ mM. A symmetric 94% specification has the complete interval

$$3.5908 \leq [B]_T/\mathrm{mM} \leq 4.1042. \tag{40}$$

At symmetric 95% fidelity, the exact binary thresholds are

$$[B]_T \geq 4.3604 \text{ mM}, \qquad [B]_T \leq 3.3710 \text{ mM}, \tag{41}$$

so the interval is empty. At $[B]_T = 7.10$ mM, the B total from the combined three-guest experiment, the central binary model predicts 90.19% C fidelity at pH 7.5 and 96.86% B fidelity at pH 12.2. These statements concern the transferred central binary model only.

At the favourable corner $(K_B^0, K_{BH^+}) = (7000, 6)$ M^{-1}, the exact binary interval is

$$6.1498 \leq [B]_T/\text{mM} \leq 6.5097. \tag{42}$$

Thus part of the full reported marginal parameter rectangle remains binary-feasible.

8 Cullen pairwise three-guest observation consistency analysis

The combined three-guest source experiment reports more than 97% cage–C and less than 2% cage–B at pH 7.5. Hence

$$\frac{X_C}{X_B} > \frac{0.97}{0.02} = 48.5. \tag{43}$$

The pairwise N-guest corollary applies even with the additional acidic guest H_2A. Since all parameter values considered satisfy $K_B^{\text{eff}} < K_C$, the endpoint factor is at most unity and

$$\frac{X_C}{X_B} \leq \frac{[C]_T K_C}{[B]_T K_B^{\text{eff}}}. \tag{44}$$

Observation consistency therefore requires

$$K_B^{\text{eff}}(7.5) < \frac{[C]_T K_C}{[B]_T 48.5} = 6.38885 \text{ M}^{-1}. \tag{45}$$

For $K_B^0 = 10^4$ M^{-1} this gives

$$K_{BH^+} < 2.4103 \text{ M}^{-1}. \tag{46}$$

Across $K_B^0 = 7000$–13000 M^{-1}, the necessary upper boundary decreases from 3.6046 to 1.2160 M^{-1}. The reported marginal K_{BH^+} range is 6–20 M^{-1}.

The deterministic rectangular box has its smallest effective middle-pH affinity at $(7000, 6)$ M^{-1}:

$$\min_{\text{box}} K_B^{\text{eff}}(7.5) = 8.78325 \text{ M}^{-1} > 6.38885 \text{ M}^{-1}. \tag{47}$$

Thus every point in the reported marginal box is incompatible with the source pairwise composition under the transferred same-site model and fixed $pK_a = 10.9$. The box is treated as a deterministic parameter rectangle; probabilistic interpretation lies outside this calculation.

9 Conditional pKa sensitivity

At the favourable box corner $K_B^0 = 7000$ M^{-1} and $K_{BH^+} = 6$ M^{-1}, observation consistency requires

$$\alpha_B < 5.5597 \times 10^{-5}. \tag{48}$$

At pH 7.5 this corresponds to

$$pK_a > 11.755. \tag{49}$$

This is approximately 0.85 units above the reported 10.9. The result should be read as a conditional sensitivity bound; an actual cage-induced pK_a shift would require independent evidence. At the central $K_{BH^+} = 13$ M^{-1}, every protonation fraction gives $K_B^{\text{eff}} \geq 13$ M^{-1} > 6.38885 M^{-1}, placing consistency beyond the reach of protonation alone.

10 Two-variable boundary construction

For two independently varied totals $u = G_{p,T}$ and $v = G_{q,T}$, the one-site model remains one-dimensional in the free host h after the two totals are fixed. Conversely, at a fixed condition and fixed h, both the host balance and any fidelity-equality condition are linear in u and v.

Let

$$a_m^x(h) = \frac{hK_m(x)}{1 + hK_m(x)}. \tag{50}$$

With fixed guest set F, host balance is

$$a_p^x(h)u + a_q^x(h)v = H_T - h - \sum_{f\in F} a_f^x(h)G_{f,T}. \tag{51}$$

A target-fidelity equality $\Phi_t(x) = \eta$ is

$$(1-\eta)X_t = \eta \sum_{m\neq t} X_m, \tag{52}$$

and is also linear in u and v at fixed h. When the target is one of the varied guests, its boundary is obtained by solving a nonsingular 2×2 system as h varies in $(0, H_T)$. For a fixed target $t \in F$, fidelity instead fixes h through $a_t^x(h)G_{t,T} = \eta(H_T - h)$, and host balance at that value gives a straight line in (u, v). The fixed-target rows are proportional and must not be inverted. Only positive points in the declared rectangle are retained. These are boundary loci where one requirement is just met; the original inequalities determine the feasible side. They do not imply convexity or reduction to one interval.

11 Full three-guest Cullen two-variable scan

The full Cullen scan fixes $H_T = 0.20$ mM and $[C]_T = 0.20$ mM, and varies $[H_2A]_T$ and $[B]_T$. The three targets are H_2A at pH 3.0, C at pH 7.5 and B at pH 12.2. Effective B affinities are calculated as in the main text. For H_2A, the neutral affinity is 2.3×10^5 M^{-1} and the charged affinity is treated as either the reported upper bound 300 M^{-1} or a favourable zero-affinity limit.

The dense two-variable scan in the domain $[H_2A]_T = 0.02$–3.0 mM and $[B]_T = 0.20$–20 mM gives the following maxima of the minimum target fidelity:

Charged H_2A affinity	Best $[H_2A]_T$ / mM	Best $[B]_T$ / mM	Maximin fidelity
300 M^{-1}	0.261	2.246	90.43%
0 M^{-1}	0.336	3.692	94.15%

At the published combined-experiment totals, $[H_2A]_T = 0.75$ mM and $[B]_T = 7.10$ mM, the target fidelities are approximately $(98.2, 77.1, 96.5)$% in the 300 M^{-1} charged-affinity model and $(98.2, 89.6, 96.9)$% in the zero-affinity limit. Thus the full three-guest concentration map strengthens the conclusion that the transferred same-site parameterization is not a quantitative reconstruction of the source mixture.

12 Naphthotube 5b/G4/G5 calculation

For the naphthotube application, the host is 5b and the two guests are G4 and G5. The association constants used are those reported by Wang and co-workers: $K_{G4}(\text{pH}7.4) = 1.7\times10^4$ M^{-1}, $K_{G5}(\text{pH}7.4) = 20$ M^{-1}, $K_{G4}(\text{pH}12) = 90$ M^{-1} and $K_{G5}(\text{pH}12) = 5.9 \times 10^3$ M^{-1}.

Using $H_T = [G4]_T = 0.10$ mM and varying $[G5]_T$, the controlled-free reference is 99.58%, while the exact finite-depletion maximin is 99.35% at $[G5]_T = 0.292$ mM. The 95%/95% interval is

$$0.0432 \leq [G5]_T/\text{mM} \leq 2.3969. \tag{53}$$

At the equimolar point $[G5]_T = 0.10$ mM, the predicted fidelities are 99.78% for G4 at pH 7.4 and 97.90% for G5 at pH 12.

13 Numerical verification protocol and edge cases

The supplementary verification script `run_analysis.py` uses NumPy's PCG64 generator with seed 24680. Positive concentrations are sampled log-uniformly over 10^{-8}–10^{-1} M for guests and 10^{-7}–10^{-1} M for host; affinities are sampled log-uniformly over 10^{-1}–10^{7} M^{-1}. The physical root is obtained with Brent's method using $h/H_T \in [0, 1]$.

Table 3: Deterministic numerical verification summary.

Verification item	Result
Factorization/product-bound cases	10,000
Maximum absolute log-factorization residual	3.56×10^{-15}
Product-bound failures	0
Closed-form threshold/root comparisons	2,000
Maximum relative threshold error	2.50×10^{-13}
Threshold decision mismatches	0
Random multiguest pairwise comparisons	5,000
Maximum absolute log pairwise-identity residual	3.56×10^{-15}
Pairwise endpoint-bound failures	0

Near-equal affinity, host-dilute, host-excess, strong-binding and weak-binding edge cases are tabulated in `generated/verification_edge_cases.csv` and in the workbook. The supplementary calculation script `run_analysis.py` records units, sampling ranges, root tolerances and generator details. These tests verify implementation; the analytical proofs provide the theorem-level justification.

14 Supplementary workbook and calculation script

The supplementary Excel workbook `finite_depletion_inverse_design.xlsx` contains the numerical data underlying the manuscript figures, including the Cullen fidelity curves, target-dependent interval bounds, parameter-plane boundaries and verification edge cases. The analysis and calculation script `run_analysis.py` is provided as a supplementary computational file for the numerical calculations and verification results.

15 Prior-art comparison

Work	Established result	Distinction of present contribution
Tight-binding/depletion literature[1, 2, 3]	Finite-total mass balances and receptor/ligand depletion	No bounded two-condition inverse specification
Wang (1995)[6]	Exact forward two-ligand total-concentration equilibrium	No bounded two-condition specification
Competitive adsorption[4, 5, 7]	Mixture isotherms and analytical finite-batch two-component solution	No fidelity/occupancy formulation window
Blay et al. (2020)[8]	Robust numerical many-ligand equilibrium and optimization	Numerical forward/optimization workflow
Present work	Closed-form bounded inverse specification	Complete fixed-one-total interval plus consistency bound

16 Hypothetical pH 12/2 design and numerical comparison

16.1 Declared inputs and interpretation

The hypothetical affinity matrix is $K_A(12) = K_A(2) = 10^4$ M^{-1}, $K_B(12) = 20$ M^{-1} and $K_B(2) = 10^4$ M^{-1}. The labels 12 and 2 identify two assumed equilibrium conditions; no protonation curve, real host, new measurement or pH-cycling kinetics is inferred from them. Fix $[A]_T = 0.20$ mM, $[B]_T \in [0.001, 20]$ mM, $\eta_a = \eta_b = 0.95$ and $\tau_a = \tau_b = 0.50$. Only H_T is adjusted, from 0.20 to 0.050 mM. All constants are assumed transferable within this hypothetical example. For a real candidate, measure effective constants under the relevant solvent, temperature and pH conditions, verify the binding stoichiometry and host stability, enter an experimentally accessible concentration domain, and apply the thresholds before selecting a recipe. Maintain the specified totals at each endpoint or recalculate for the actual dilution. The feasible interval describes the predicted equilibrium endpoint compositions.

At pH 2 the two affinities are equal, so the bound A/B ratio equals the total A/B ratio even under depletion. Therefore the lower fidelity threshold is exactly $19[A]_T = 3.8$ mM for both host totals. At pH 12, A binds much more strongly than B; increasing host total depletes free A disproportionately. This lowers the upper B threshold to 2.682638 mM at $H_T = 0.20$ mM and makes the two-condition intersection empty. Reducing H_T to 0.050 mM weakens this depletion and raises the upper B threshold to 4.463624 mM. The occupancy lower bounds are zero because A alone already meets the 50% occupancy floor at both host totals; the floor remains an independent specification in the calculation. The non-depleting selectivity swing is $S = 500$ and the controlled-free reference is $\sqrt{500}/(1 + \sqrt{500}) = 0.9571930265$. This reference does not certify finite-total feasibility.

16.2 Independent numerical equilibrium and threshold calculations

The new script `revision_analysis.py` uses only the Python standard library. Its numerical branch solves the original host balance in $z = h/H_T \in [0, 1]$ by bracketed bisection. Bound concentrations and all metrics are then computed from mass action. A separate outer bisection in $[B]_T$ (scaled to mM) locates each fidelity crossing on $[0, 20]$ mM; an occupancy threshold is set to zero only if its floor is already met at zero B. The numerical branch does not call the analytic threshold expressions. Bisection stops at adjacent floating-point endpoints, at an exact floating-point zero, or after 100 iterations. The two methods therefore use the same model, inputs and domain. Their agreement validates the calculation, while the monotonicity proof supplies the completeness statement for either method.

Table 4: Analytic versus independently located numerical thresholds. All concentrations are mM. Zero denotes a floor already met at zero B.

H_T	Threshold	Closed form	Numerical
0.200	pH 2 fidelity lower	3.800000000	3.800000000
0.200	pH 12 fidelity upper	2.682637630	2.682637630
0.200	pH 12 occupancy lower	0.000000000	0.000000000
0.200	pH 2 occupancy lower	0.000000000	0.000000000
0.050	pH 2 fidelity lower	3.800000000	3.800000000
0.050	pH 12 fidelity upper	4.463624033	4.463624033
0.050	pH 12 occupancy lower	0.000000000	0.000000000
0.050	pH 2 occupancy lower	0.000000000	0.000000000

16.3 Finite grids and endpoint classifications

Uniform grids include both endpoints of $[0.001, 20]$ mM and use $B_k = L + k(U - L)/(N - 1)$, $k = 0, \ldots, N - 1$. Every point is classified by two forward equilibrium solves and all four performance inequalities. The same solver is used for every grid resolution; grid points are not chosen from the analytic boundaries. The 8-point grid misses the adjusted feasible interval, whereas finer grids find interior recipes. A sampled range is not the exact boundary interval.

Table 5: Finite uniform-grid comparison. Concentrations and step sizes are mM. The sampled range is the smallest through largest passing grid value.

H_T	N	Step	Passing	Sampled range
0.200	8	2.857000	0	none
0.200	21	0.999950	0	none
0.200	201	0.099995	0	none
0.200	2001	0.009999	0	none
0.050	8	2.857000	0	none
0.050	21	0.999950	1	4.000800–4.000800
0.050	201	0.099995	7	3.800810–4.400780
0.050	2001	0.009999	67	3.800810–4.460777

At the candidate $[B]_T = 4.000$ mM and $H_T = 0.050$ mM, $\Phi_A(12) = 95.4935\%$, $\Phi_B(2) = 95.2381\%$, $\theta(12) = 63.9586\%$ and $\theta(2) = 97.6477\%$. The desired-complex yields $Y_A(12) = 61.0764\%$ and $Y_B(2) = 92.9978\%$ are reported separately. The complete equilibrium concentrations are supplied in `revision_example_summary.json`.

Table 6: Independent classification at and around the adjusted interval. The two outside checks are 0.001 mM beyond the respective boundaries. Both occupancy floors are met in all five rows. Percentages are conditional fidelities; endpoint comparisons allow 5×10^{-12} numerical roundoff.

Point	$[B]_T$ (mM)	$\Phi_A(12)$ (%)	$\Phi_B(2)$ (%)	All targets
below lower	3.799000	95.70927	94.99875	no
lower endpoint	3.800000	95.70820	95.00000	yes
candidate	4.000000	95.49354	95.23810	yes
upper endpoint	4.463624	95.00000	95.71149	yes
above upper	4.464624	94.99894	95.71241	no

The script verifies threshold agreement to 10^{-10} M, mass-balance residuals, the equal-affinity identity at pH 2, both binding endpoints, interior and exterior classifications, and agreement of analytic membership with 257 additional forward-solved concentrations for each host total. The measured discrepancies and pass/fail status are recorded in `revision_verification.json`. The generated CSV files preserve all reported thresholds, grid results and point classifications.

References

[1] J. F. Morrison, *Biochim. Biophys. Acta*, 1969, **185**, 269–286, DOI: 10.1016/0005-2744(69)90420-3.

[2] P. J. F. Henderson, *Biochem. J.*, 1972, **127**, 321–333, DOI: 10.1042/bj1270321.

[3] K.-J. Chang, S. Jacobs and P. Cuatrecasas, *Biochim. Biophys. Acta*, 1975, **406**, 294–303, DOI: 10.1016/0005-2736(75)90011-5.

[4] I. Langmuir, *J. Am. Chem. Soc.*, 1918, **40**, 1361–1403, DOI: 10.1021/ja02242a004.

[5] E. C. Markham and A. F. Benton, *J. Am. Chem. Soc.*, 1931, **53**, 497–507, DOI: 10.1021/ja01353a013.

[6] Z.-X. Wang, *FEBS Lett.*, 1995, **360**, 111–114, DOI: 10.1016/0014-5793(95)00062-E.

[7] N. Kaiblinger, R. Hahn, J. Beck, Y. Wang and G. Carta, *Adsorption*, 2024, **30**, 51–56, DOI: 10.1007/s10450-023-00429-4.

[8] V. Blay, I. Otero-Muras and D. A. Annis, *Anal. Chem.*, 2020, **92**, 12630–12638, DOI: 10.1021/acs.analchem.0c02715.

[9] W. Cullen, S. Turega, C. A. Hunter and M. D. Ward, *Chem. Sci.*, 2015, **6**, 625–631, DOI: 10.1039/C4SC02090A.

[10] W. Cullen, K. A. Thomas, C. A. Hunter and M. D. Ward, *Chem. Sci.*, 2015, **6**, 4025–4028, DOI: 10.1039/C5SC01475A.

[11] X. Wang, M. Quan, H. Yao, X.-Y. Pang, H. Ke and W. Jiang, *Nat. Commun.*, 2022, **13**, 2291, DOI: 10.1038/s41467-022-30012-4.
